# A century of coping with environmental and ecological changes via compensatory biomineralization in mussels

**DOI:** 10.1111/gcb.15417

**Published:** 2020-11-21

**Authors:** Luca Telesca, Lloyd S. Peck, Thierry Backeljau, Mario F. Heinig, Elizabeth M. Harper

**Affiliations:** ^1^ Department of Earth Sciences University of Cambridge Cambridge UK; ^2^ British Antarctic Survey Cambridge UK; ^3^ Royal Belgian Institute of Natural Sciences Brussels Belgium; ^4^ Evolutionary Ecology Group University of Antwerp Antwerp Belgium; ^5^ Technical University of Denmark DTU Nanolab National Centre for Nano Fabrication and Characterization Kongens Lyngby Denmark

**Keywords:** biomineralization, climate change, compensatory mechanisms, foundation species, multiple stressors, museum collections, *Mytilus*, resistance

## Abstract

Accurate biological models are critical to predict biotic responses to climate change and human‐caused disturbances. Current understanding of organismal responses to change stems from studies over relatively short timescales. However, most projections lack long‐term observations incorporating the potential for transgenerational phenotypic plasticity and genetic adaption, the keys to resistance. Here, we describe unexpected temporal compensatory responses in biomineralization as a mechanism for resistance to altered environmental conditions and predation impacts in a calcifying foundation species. We evaluated exceptional archival specimens of the blue mussel *Mytilus edulis* collected regularly between 1904 and 2016 along 15 km of Belgian coastline, along with records of key environmental descriptors and predators. Contrary to global‐scale predictions, shell production increased over the last century, highlighting a protective capacity of mussels for qualitative and quantitative trade‐offs in biomineralization as compensatory responses to altered environments. We also demonstrated the role of changes in predator communities in stimulating unanticipated biological trends that run contrary to experimental predictive models under future climate scenarios. Analysis of archival records has a key role for anticipating emergent impacts of climate change.

## Graphical abstract

Current understanding of organismal responses to change stems from relatively short‐term studies. Most projections lack long‐term observations incorporating the potential for transgenerational phenotypic plasticity and genetic adaption. We describe unexpected temporal responses in biomineralization as a mechanism for resistance to altered environments in the blue mussel *Mytilus edulis*. We evaluated archival mussel specimens collected regularly between 1904 and 2016, along with records of environmental descriptors and predators. Contrary to global‐scale predictions, shell production increased over the last century, highlighting a protective capacity of mussels for qualitative and quantitative trade‐offs in biomineralization as compensatory responses to altered environments and predator communities.

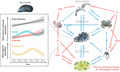

## 1 INTRODUCTION

As global climate change accelerates (Kirtman et al., 2013), we need accurate predictions about biological responses if we are to prevent emergent damage to the biosphere (Nagelkerken & Connell, 2015; Urban et al., 2016). However, our ability to project changes to individual species and populations, let alone communities and ecosystems, in response to disturbances is limited (Kroeker et al., 2017; Urban et al., 2016). The overwhelming majority of models forecasting biotic change stem from experimental observations on relatively short timescales (Kroeker et al., 2013; Nagelkerken & Connell, 2015). Long‐term data are lacking in natural systems incorporating the role of phenotypic plasticity, both within and across generations, in combination with long‐term genetic adaption in populations, the processes identified as critical for responding to altered environments (Cross et al., 2018; Thomsen et al., 2017; Vargas et al., 2017). Thus, a mechanistic understanding of processes shaping biotic variation in functioning ecosystems, across relevant timescales to evaluate potential impacts of long‐term acclimation and adaption, is critical for building the theoretical framework necessary to anticipate the scope of biological responses (Kroeker et al., 2017; Urban et al., 2016).

Compensatory mechanisms are important mediators of biotic dynamics with the potential to attenuate (Ghedini et al., 2015), or even reverse (Connell et al., 2017), the direct impacts of change on overall performance and ecological functions of organisms (Cross et al., 2019; Ghedini et al., 2015). Compensation can arise from a range of physiological, habitat, and ecological alterations acting at organismal, population (Ashton et al., 2017; Cross et al., 2019), or community level (Ghedini et al., 2015; Peck et al., 2015). These biological mechanisms have, however, seldom been examined in species with both high climate sensitivity and disproportionate ecological impact on healthy ecosystem functioning (McCoy & Ragazzola, 2014; Telesca et al., 2019; Urban et al., 2016), such as calcifying foundation species.

Climate change has caused major alterations in ocean physics and chemistry (Kirtman et al., 2013). These changes, which profoundly affect marine community structure and ecosystem dynamics, have been further exacerbated in nearshore habitats by increasing human pressures over the last century (Nagelkerken & Connell, 2015; Urban et al., 2016). Most environmental change research on marine calcifiers has focused on early life‐history stages given the expectation, and largely the findings, that intensifying climate stressors act more strongly on larvae and juvenile stages than adults (Gazeau et al., 2013; Kroeker et al., 2013; Waldbusser et al., 2015). Such projections about calcifiers' susceptibility to change predominantly originate from short term to long term, from days to 1 or 2 years, laboratory (Kroeker et al., 2013; Nagelkerken & Connell, 2015), mesocosm and in situ manipulations (Ashton et al., 2017; Connell et al., 2017; Peck et al., 2015). These studies on simplified experimental ‘communities’, despite being long term for experiments, may not translate to very long‐term population dynamics within real‐world naturally complex ecological systems (Connell et al., 2017; Peck et al., 2015; Vargas et al., 2017). Recent in situ works have begun to address this problem (Ashton et al., 2017; Clark et al., 2019), but a rarely used approach to evaluate longer‐term responses, from years to decades, and the target of potential population‐level selection involves using archival collections (Cross et al., 2018; McCoy & Ragazzola, 2014; Pfister et al., 2016). Although historical studies on archival material are correlative in their nature and might, therefore, be constrained by the limited parallel records of environmental information at the corresponding timescale of the biological processes analysed, they are especially useful to (a) assess ecological effects of stressors as they are introduced over climate change relevant timescales (McCoy & Ragazzola, 2014); and (b) include evaluation of population responses via transgenerational phenotypic plasticity and possible long‐term adaption, both keys to resistance (Thomsen et al., 2017; Vargas et al., 2017), complementing experimental data but not incorporated in any other approach (Cross et al., 2018; Vargas et al., 2017).

**FIGURE 1:**
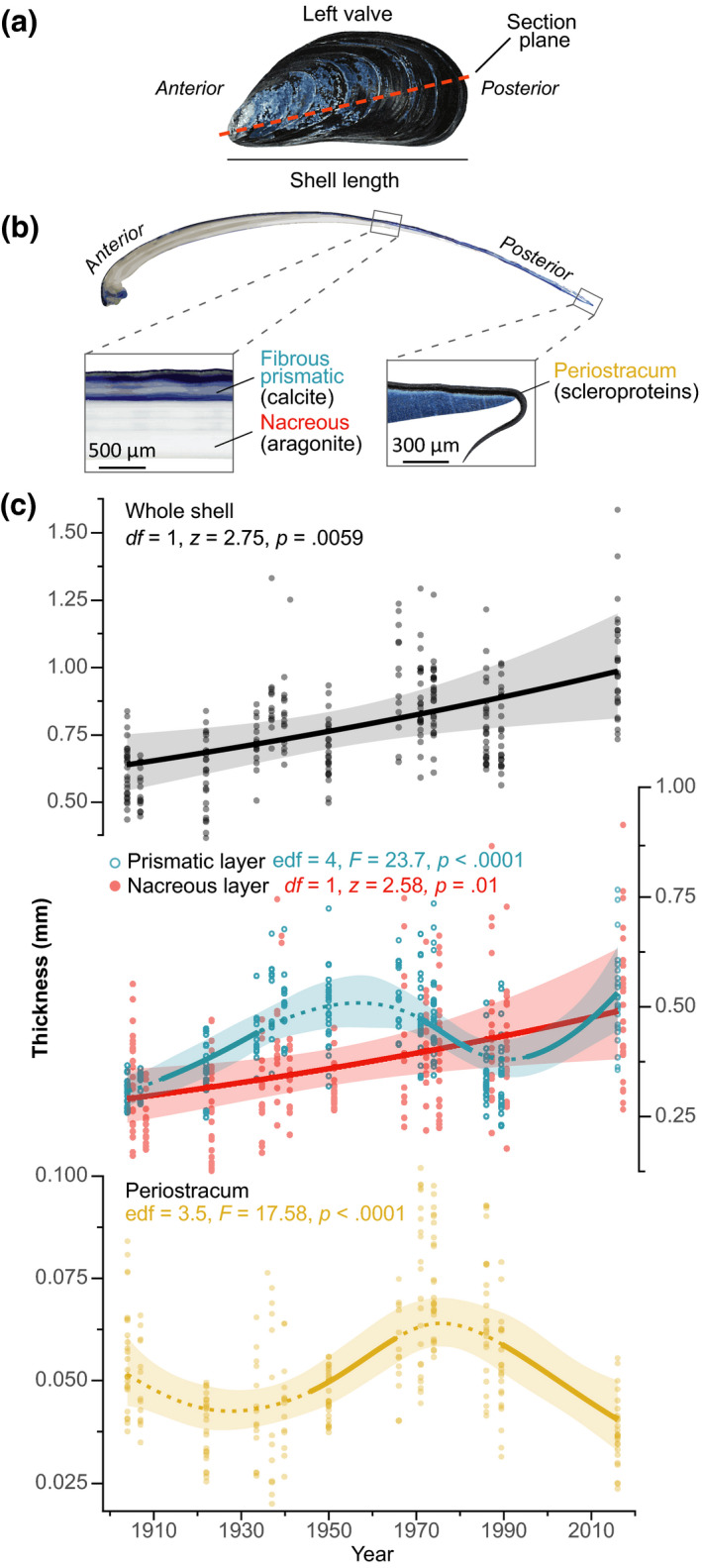
Historical patterns of blue mussel shell thickness along the Belgian coast between 1904 and 2016. (a) *Mytilus* *edulis*shell morphology and structure. (b) Antero‐posterior cross‐section of a left valve showing the arrangement of shell layers. The shell consists of three layers: (i) the outer periostracum; (ii) the fibrous prismatic layer; and (iii) the nacreous layer. The organic periostracum is made of scleroproteins (quinone‐tanned). The calcified fibrous prismatic and nacreous layers are mainly composed of calcium carbonate (CaCO_3_) crystals of different mineral forms, calcite and aragonite, respectively, and minor amounts of inter‐ and intra‐crystalline organic matrix. (c) Predicted temporal trends of shell layer thicknesses for the mean shell length of the sample (53.13 mm) between 1904 and 2016. Whole‐shell and nacreous layer thickness increased linearly over time. Prismatic layer deposition significantly decreased between 1970 and 1983, and increased in the 1911–1934 period and after 1995. Periostracum thickness increased between 1945 and 1962, and decreased by 29% decrease after 1984. Simultaneous 95% confidence intervals (shaded areas) and periods of significant change (solid lines) are reported

Marine mussels of the genus *Mytilus* are bed‐forming foundation species throughout the world's eulittoral ecosystems. Owing to their economic value, ecosystem services provided, and projected vulnerability (Gazeau et al., 2013; Thomsen et al., 2017), mytilids have received much attention as key indicator species (Kroeker et al., 2016; Telesca et al., 2018, 2019). However, because of the ubiquity of mussels, extensive museum collections of *Mytilus* from the last century are exceptionally rare. Calcareous mussel shells (Figure 1a,b) perform several vital functions including structural support and protection from predators. Because shell integrity determines survival, shell traits, and their resulting mechanical properties are subject to strong selection pressure, with functional success or failure a fundamental evolutionary driving force (Freeman, 2007; Watson et al., 2017). Thus, an understanding of potential compensatory mechanisms in shell biomineralization to withstand environmental insult and altered predation impacts is essential to inform realistic projections of calcifiers' persistence and ecological functions when selection pressures alter in changing environments.

Here, we describe temporal changes in biomineralization in the blue mussel *Mytilus edulis* using a unique museum collection of specimens sampled from intertidal breakwaters along 15 km of Belgian coastline at near decadal frequency between 1904 and 2016. By combining extensive long‐term datasets of key environmental descriptors and predators in a naturally controlled system, across a centennial timescale incorporating long‐term acclimation and adaption, we describe (a) compensatory trade‐offs in shell biomineralization as potential mechanism for resistance to altered environments and species interactions; and (b) the role of changes in predator communities in stimulating unanticipated trends in biomineralization that run contrary to global‐scale projections under future climate scenarios.

## 2 MATERIALS AND METHODS

### 2.1 Museum shell collections

We evaluated 268 specimens of the blue mussel, *M. edulis*, collected with an almost decadal frequency between 1904 and 2016 along the Belgian coastline at 13 sites between Oostende (51°14′16.27″N–2°55′03.09″E) and Nieuwpoort (51°09′14.14″N–2°43′23.62″E; see Figure S1), a total distance of 15 km. Collection details are provided in Table S1.

Specimens collected between 1904 and 1987 were obtained from archival shell collections of the Royal Belgian Institute of Natural Sciences (RBINS). This unique collection of a single species is composed of both wet (shells and body tissue preserved in 70% ethanol) and dry (shells only) specimens that were obtained from monitoring programmes during the 20th century. Only archival specimens with a well‐preserved periostracum and no evident deterioration of internal calcareous shell layers, as well as with information on species identity, collection date and location were evaluated. Only general information on the tidal ranges of specimens (i.e. intertidal or subtidal) were reported in collection notes for most of the sampling sites; therefore, exact tidal height could not be obtained. To minimize the potential influence of differences in tidal habitats on mussels shell formation (i.e. differences in growth rate; Seed, 1969), we selected adult *M. edulis* with shell lengths of 39–66 mm, collected from intertidal stone breakwaters. Specimens of this size class were selected to (a) avoid bias in the analyses due to the use of shells with different size ranges for each collection site; and (b) control statistically for the effect of shell size variations on shell layers deposition. In all, 30 intertidal adults (shell length of 44–62 mm) were hand‐collected in 2016 from a single location and substratum (intertidal stone breakwaters in Mariakerke). For each specimen, shell length was measured with digital calipers (0.01 mm precision) and used to control for within‐year shell size differences (Telesca et al., 2018). Animal age was estimated by counting annual shell increments. Because of the limited control we had on the selection of specimens from specific intertidal heights, we tested for age differences in mussels of similar shell size between collection sites/years (indicative of potential differences in growth rate due to habitat heterogeneity).

### 2.2 Shell preparation and layer measurement

We set left shell valves (*n* = 256) in polyester resin (Kleer‐Set FF, MetPrep). Embedded specimens were sliced longitudinally along their axis of maximum growth (Figure 1a,b) using a diamond saw and then progressively polished with silicon carbide paper (grit size: P800‐P2500) and diamond paste (grading: 9–1 µm). Shell thickness was measured on polished cross‐sections photographed with a stereomicroscope (Leica M165 C equipped with a DFC295 HD camera; Leica). Because many shells had undergone evident environmental abrasion, which removed the periostracum and prismatic layer near the anterior shell side (umbo), we estimated whole‐shell, prismatic, and nacreous layers thicknesses at the midpoint along the shell cross‐section (Telesca et al., 2019). Periostracum thickness was measured at the posterior edge where it attaches to the external surface of the prismatic layer. In this way, we estimated the fully formed organic layer that was unaffected by decay and abrasion caused by physical‐environmental agents during shell growth or potential deterioration (detachment) due to preservation (Harper, 1997; Figure 1b).

### 2.3 Organic content analysis

We performed thermogravimetric analyses (TGA) to estimate the weight proportion (wt%) of organic matrix within the prismatic layer. *M. edulis* specimens were selected from 3 years, 1904, 1950, and 2016 (*n* = 14, 14, and 15, respectively), to explore differences in shell organic content. The periostracum was removed by sanding, and prismatic layer tiles (8 × 5 mm, *n* = 43) were cut along the posteroventral shell margin, cleaned, air‐dried, and then finely ground. We tested 10 mg of this powdered shell with a thermogravimetric analyzer (TGA Q500; TA Instruments). Samples were subjected to constant heating from ~25°C to 700°C at a linear rate of 10°C/min under a dynamic nitrogen atmosphere and weight changes were recorded (see Methods S1). We estimated the wt% of organic matter within the shell microstructure as weight loss percent during the thermal treatment between 150°C and 550°C (Figure S2).

### 2.4 Elliptic Fourier analysis of shell outlines

Shape analyses of *M. edulis* shell valves were carried out through a geometric morphometrics approach using an elliptic Fourier analysis (EFA) of outlines. Outline processing and EFA were carried out using the package Momocs (Table S2) in the software R (v3.5.2; R Core Team, 2018).

Outlines of left shell valve lateral views (*n* = 268) were digitized, processed, and analysed with an EFA following Telesca et al. (2018; see Methods S1). We chose seven harmonics, encompassing 99% of the total harmonic power (Figure S3). Four coefficients per harmonic (28 descriptors) were extracted for each shell outline and used as variables quantifying geometric information.

A principal component analysis (PCA) was performed on the harmonic coefficients to define axes capturing most of the shape variation among individuals. Principal components (PCs) were considered as new shape variables. To understand the contribution of harmonic coefficients to shell shape, we reconstructed extreme outlines along each PC (Figures S3 and S4). The first seven PCs (99% of outline variation) were analysed with a MANOVA to test for significant effects of collection year and shell length (size) on shape variances (Figure S4). To visualize outline differences, we generated deformation grids and iso‐deformation lines through mathematical formalization of thin plate splines analysis (Figure S3).

### 2.5 Environmental dataset

To understand temporal changes in marine conditions along the Belgian coast, we evaluated available long‐term datasets of key environmental descriptors for the collection location: sea surface temperature (SST), sea surface salinity (SSS), chlorophyll‐*a* (Chl‐*a*) concentration (as a proxy for food supply; Thomsen et al., 2013) and dissolved oxygen (see Data S1). Key descriptors were selected based on known influences on mussel growth, their availability for the sampling location, and forecasted ocean alterations under climate change (Kirtman et al., 2013).

Time series of daily measurements for the period 1900–1984 in the collection area 51°14′N–51°05′N, 02°55′E–02°32′E were obtained from the International Council for the Exploration of the Sea (ICES), the Integrated Marine Environmental Readings and Samples, and the Management Unit of the Mathematical Model of the North Sea Datasets (MUMM at RBINS). Environmental datasets for 1985–2016 were generated using MUMM data and the Copernicus Marine Environment Monitoring Service (Data S1). These climate data, comprising high‐resolution physical and biogeochemical assimilated daily observations, presented several advantages compared to traditional measurements from their high spatiotemporal resolution, both coverage and frequency (continuous daily measurements across 31 years), advanced calibration and validation (Telesca et al., 2018, 2019). Other parameters and local process with a potential influence on mussel biomineralization could not be included due to their limited availability or absence of historical datasets for the collection sites.

### 2.6 Mussel‐predators dataset

To describe historical changes in predation regime and pressure on intertidal mussel beds and their shell traits, we analysed long‐term abundance datasets of key intertidal predators with a potential influence on mussel shell traits: decapods (benthos and planktonic larvae), seagulls and dog whelks. We selected key predator taxa based on historical dataset availability for the study location, their predation strategy (i.e. durophagy and drilling), and known effects on shell biomineralization responses in *Mytilus* spp. (Freeman, 2007; Lowen et al., 2013; Sherker et al., 2017).

Information on macrobenthic decapod abundances (number of individuals per sample) for the 1978–2017 in the Belgian coastal area (51°20′N–51°05′N, 03°12′E–02°32′E) were obtained from the ICES Data Centre, MUMM Datasets, and the European Environment Agency (EEA), including analyses based on fisheries data, coastal benthos survey projects, and literature. Decapod larvae datasets for 1958–2009 were obtained from ICES survey data and EEA. Numbers and location of breeding pairs of the four dominant seagull species (*Larus ridibundus*, *L. fuscus graellsii*, *L. argentatus*, and *L. canus*), along the Belgian coastline (Zeebrugge–Middelkerke) for 1960–2007 were obtained from literature. Local reports were used to assess the presence of the dog whelk, *Nucella lapillus*, along the Belgian shores (see Data S1).

The predatory starfish *Asterias* spp. was excluded from our study due to (a) the absence of historical datasets for the study location; (b) the reported changes in echinoderm abundances and ranges at a North Sea scale after the mid‐1980s mainly regard echinoids and ophiuroids (Kirby & Beaugrand, 2009); (c) their limited influence on intertidal mussel compared to subtidal mussel beds (Seed, 1969; Seed & Suchanek, 1992); and (d) the inducible defence response in *Mytilus* spp. shells of this predator that pulls the shells apart, including development of stronger adductor muscles, increased byssal strength, and occasional trade‐offs between growth and shell thickness with no net effect on shell deposition (shell weight; Freeman, 2007; Lowen et al., 2013).

### 2.7 Statistical analysis

Generalized additive mixed‐models (GAMMs; Wood, 2017) were used to describe long‐term variations in environmental descriptors and predator abundances, as well as temporal trends in mussel biomineralization and shell shape. GAMMs allowed us to (a) account for the hierarchical structure of the datasets (multiple *M. edulis* specimens from each collection site; *n* = 12–30); (b) specify flexible dependence structures of the response on the covariates; and (c) control for variations (‘noise’) among sampling locations (Bolker, 2015).

We carried out data exploration following the protocol of Zuur et al. (2010). Initial inspection revealed no outliers. Pairwise scatterplots and variance inflation factors (VIFs) were calculated to check for collinearity between input variables. VIF < 2 indicated an acceptable degree of correlation among covariates within the same model. Preliminary inspection of residual patterns showed heteroscedasticity in most models. This required using different combinations of link functions and probability distributions, allowing greater variation for large mean values (i.e. gamma or inverse Gaussian for continuous data, Poisson or negative binomial for count data) for individual responses. When changing the underlying distribution did not stabilize the variance, a ln‐transformation of continuous response variables (excluding proportions) was required.

### 2.8 Time‐series analysis

We used GAMMs to model long‐term and seasonal changes in time series of environmental descriptors and the abundance of predators. Time series, consisting of discontinuous daily observations per year, were expressed as continuous monthly averaged measurements. Fixed covariates were *month* (seasonal variation, expressed as numeric 1, …, 12 indicator), *year* (trend), both fitted as smoothers, and *month* × *year* interaction that was defined through a smooth tensor product interaction (Wood, 2017). The tensor product interaction is used to represent functions of covariates, which are measured in different units (i.e. different magnitude of change), by allowing the smooth effect of one variable (seasonal variation) to vary as a smooth function of the second variable (trend). This allowed the within‐year spline (effect) to change smoothly with the between‐year effect.

(1) $$\begin{array}{cc} & Series_{i}=\beta_{0}+f_{\text{seasonal}}(Month_{i})+f_{\text{trend}}(Year_{i})+f(Month_{i},\;Year_{i})+\varepsilon_{i}\\ & \;\varepsilon_{i}∼N(0;\;\sigma^{2}\Lambda ), \end{array}$$

where *Series_i_* is our *i*th observation of the time series, *β*_0_ is the intercept, *f*_seasonal_ and *f*_trend_ are smooth functions for the seasonal and trend features of interest, *f* is the smooth function (interaction) of our two time variables, and *ε*_i_ the normally distributed and correlated (**Λ**) error. By allowing the seasonal component to change in time along with the trend, we modelled (a) any seasonal, or within‐year variation; (b) any trend, or long‐term change, in the mean level of the time series; and (c) any interaction between seasonal and trend features of the data.

Boundaries and numbers of knots (limits and dimensions of the basis used for splines) were manually selected while effective degrees of freedom were estimated by the smooth function (Wood, 2017). A cubic regression spline basis was used for the trend smooth term, whereas a cyclic cubic spline basis was used for the seasonal smooth term to avoid discontinuity between January and December values (Wood, 2017). Models indicated significant within‐year residuals autocorrelation, which required the use of different residual correlation structures (Wood, 2017). Autoregressive (for equally spaced time series) or conditional autoregressive (for unequally spaced observations) models, nested within each *year*, were fitted to the residuals to account for temporal autocorrelation.

A negative binomial generalized additive model with a log link function was used to model numbers of seagull breeding pairs as a function of year and to compare between the four species. The log link function ensures positive fitted values, and the negative binomial distribution is typically used for count data that are overdispersed with respect to a Poisson distribution. Covariates were year (smoother with a cubic spline), gull species (categorical, four levels: seagull species), and their interaction.

### 2.9 Statistical modelling

Separate GAMMs were used to describe shell thickness (*n* = 256) and shape (*n* = 268) with respect to time, and to compare between shell measurements (thickness of whole‐shell, periostracum, prismatic, and nacreous layers) and morphological traits (PC1 and PC2 from EFA coefficients). Fixed covariates were collection *year* and shell *length* (both fitted as smoothers). To estimate biologically meaningful intercepts, we centred the input variables. Collection *year* was centred to the year 1900, while *length* to sample mean shell length (53.13 mm). Shell *length* was included in all models to control for the effect of shell size variations on shell measurements and morphological traits. To incorporate dependency among observations from the same sampling site, we used *site* as a random intercept.

(2) $$\begin{array}{cc} Response_{\mathrm{ij}} & =\beta_{0}+f_{1}(Year_{i})+f_{2}(Length_{\mathrm{ij}})+Site_{i}+\varepsilon_{\mathrm{ij}}\\ Site_{i} & ∼N\left(0;\sigma_{\mathrm{Site}}^{2}\right)\\ \varepsilon_{\mathrm{ij}} & ∼N(0;\sigma^{2}), \end{array}$$

where *Response_ij_* is the *j*th observation for the response variable (thickness or shape variable) in site *i* (*i* = 1, …, 13), *f* is the smoothing function (cubic regression spline), and *β*_0_ is an intercept. *ε_ij_* is the noise and *Site_i_* is the random intercept, which are assumed to be independently distributed with expectation 0 and variance *σ* ^2^ and $\sigma_{\mathrm{Site}}^{2}$, respectively.

We modelled the wt% of organic matrix (*n* = 43) as a function of year of collection (categorical, three levels: 1904, 1950, and 2016) and prismatic layer thickness (continuous), to test for differences among time periods and association with shell deposition. The response variable was coded as a value from 0 to 1; therefore, a generalized linear model with a beta distribution and a logistic link function was used. Pairwise contrasts with a standard Bonferroni correction were then used to test (*α* = 0.017) for differences in the wt% of organics among collection years.

To estimate potential interactions of local environmental conditions on mussel shell biomineralization, we first calculated descriptive statistics for individual factors per year (i.e. mean, median, standard deviation, 10th, 25th, 75th, and 90th percentiles, minimum and maximum annual values). To provide a first‐order approximation of the water conditions during the lifespan of sampled specimens, we expressed the descriptive statistics as average values for the 6‐year period prior to collection (4 years for individuals from 1904). We then standardized all the descriptors to account for differences in measurements units by subtracting the sample mean from the variable values and dividing them by the sample *SD* [*z_i_* = (*x_i_* − $\bar{x}$)/*σ_x_*]. Next, we performed separate PCAs on descriptive statistics for SST and SSS to create PCs, characterizing SST and SSS ‘regimes’ (Kroeker et al., 2016). We then used the first two PCs scores from each PCA as input variables (Table S3), along with PCs from EFAs (shape‐PCs; Figure S4) included as proxies for food supply (Telesca et al., 2018), as fixed covariates (smoothers) in GAMMs to model variations in shell thickness measurements. Collection site was included as a random intercept.

To describe variations of shell traits under changing predation regimes, we modelled thickness of each shell layer as a function of predators' abundance (low/high: decapods and gulls) and presence (presence/absence: dog whelks). Thickness measurements were selected depending on the temporal availability of predator datasets. Individual layers were compared between conditions (treatment) for each predator type: decapods (low: 1970–1988; high: 1989–2016), gulls (low: 1980–1990; high: 1991–2016), and dog whelk (presence: before 1981; absence: after 1981).

### 2.10 Model optimization and predictions

Models were optimized by first selecting the random structure and then the optimal fixed component. The principal tools for model comparison were the corrected Akaike information criterion (AICc) and likelihood ratio tests. Random terms were selected on prior knowledge of the dependency structure of the dataset. Visual inspection of residual patterns indicated violation of homogeneity in most cases. This required the use of variance structures (generalized least squares) allowing the residual spread to vary with respect to predictors. To assess the presence of temporal dependence in model residuals, we used (partial) autocorrelation functions, for regularly spaced time series, or variograms, for irregularly space ones. When temporal autocorrelation was found, the best residual correlation structure was selected through comparisons of models with differing stochastic trend models in the residuals. The fixed component was optimized by rejecting non‐significant interaction terms only that minimized the AICc value. For environmental GAMMs with multiple smooth terms, selection was carried out using cubic regression splines with shrinkage (Wood, 2017). Final models were validated by inspection of standardized residual patterns to verify assumptions of normality, homogeneity, and independence. The proportion of variance explained by the models was quantified with conditional determination coefficients (c*R* ^2^).

Optimal models were used to describe changes in mussel shell characteristics over the last century. All predictions for temporal changes of shell thickness and morphological traits were computed controlling statistically for among‐individual variations in shell length. We used a simulation‐based approach to identify periods of statistically significant change in the time series (see Methods S1; Figure S5). This information was used to identify which parts of the predicted trend were significantly increasing or decreasing over time. All data exploration and statistical analyses were performed with the software R (Table S2).

## 3 RESULTS

### 3.1 Historical patterns of shell deposition and calcification

Shell deposition increased by 57% between 1904 and 2016 (*z*_251_ = 2.75, *p* = .0059, *n* = 256 shells), with prismatic and nacreous layers from modern shells (2016) being on average 48% and 74% thicker for given sized shells (prismatic: *F*_4,250_ = 23.7, *p* < .0001; nacre: *z*_251_ = 2.58, *p* = .01; Table S4), respectively, than those of the oldest archival specimens (Figure 1c). Periostracum thickness changed nonlinearly over the last 112 years (*F*_3.5,250_ = 17.58, *p* < .0001; Figure 1c).

**FIGURE 2:**
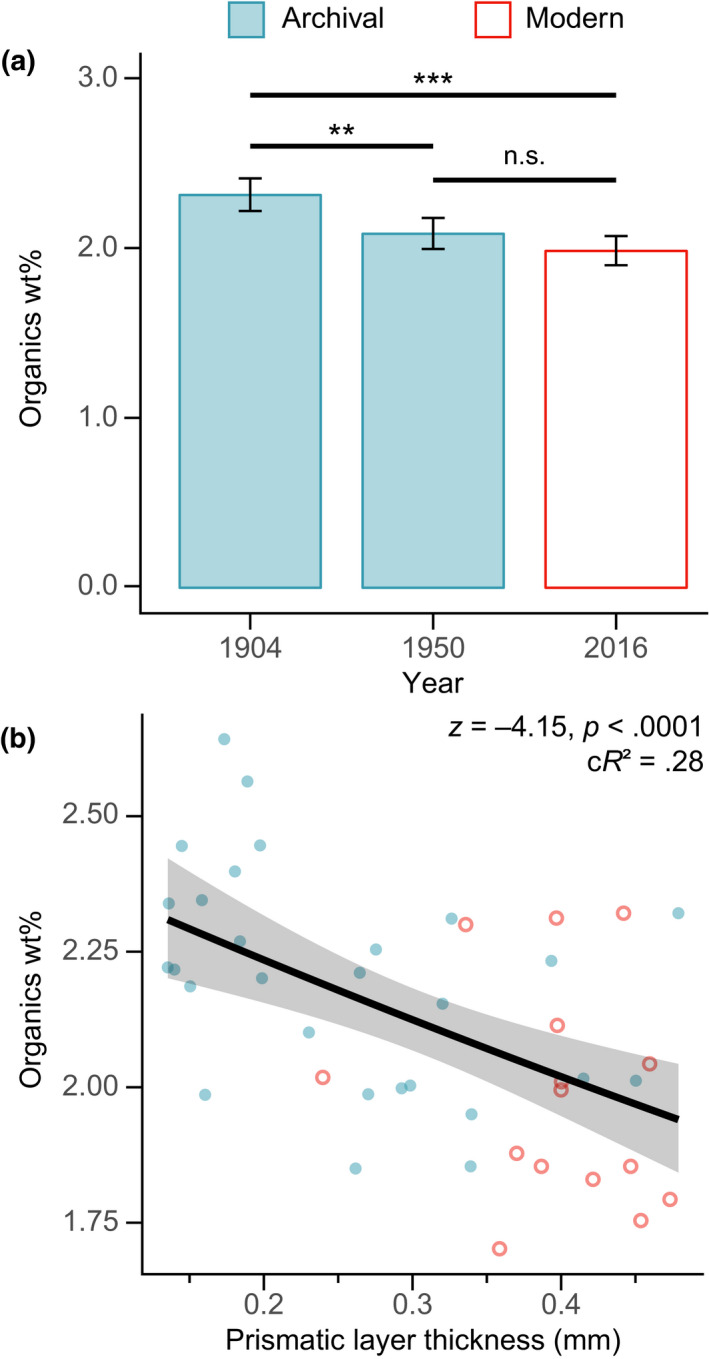
Temporal variations of shell organic content and calcification. (a) Variations in organic content of blue mussel shells indicating significantly lower proportions of organic matrix in modern (red bar) than archival (blue bars) specimens (***p < .0001), a significant difference in organics between 1904 and 1950 (0.11 wt%; *z*_39_ = −3.38, *p* = .00073[**]) and no significant difference between 1950 and 2016 (0.05 wt%; *z*_39_ = 1.58, *p* = .12[n.s.]). (b) Negative relationship between the wt% of organics and prismatic layer thickness, suggesting a positive association between calcification level (wt% of CaCO_3_) and layer thickness

Thermogravimetric analyses indicated the weight proportion (wt%) of organic content within the prismatic layer of modern mussels (2016; mean wt% [*SD*] = 1.99 [0.20]) was 14% lower (larger wt% of CaCO_3_) than that of archival shells (1904; mean wt% [*SD*] = 2.31 [0.18]; *z*_39_ = 5.02, *p* < .0001, *n* = 43; Figure 2a). Organic content significantly decreased with increasing shell layer thickness (*z*_40_ = −4.15, *p* < .0001, *n* = 43; Figure 2b), indicating that *M. edulis* produced more calcified and thicker shells over time.

An EFA no clear trend in shell shape between 1904 and 2016 (shape‐PC1: *F*_5,260_ = 2.86, *p* = .12 and shape‐PC2: *F*_5,260_ = 1.10, *p* = .32; *n* = 265; Figures S3 and S4; Table S4). No significant difference in animal age (number of annual shell increments) between collection years for individuals of same shell size was detected (*F*_13,247_ = 1.85, *p* = .28).

### 3.2 Long‐term changes in environmental conditions

**FIGURE 3:**
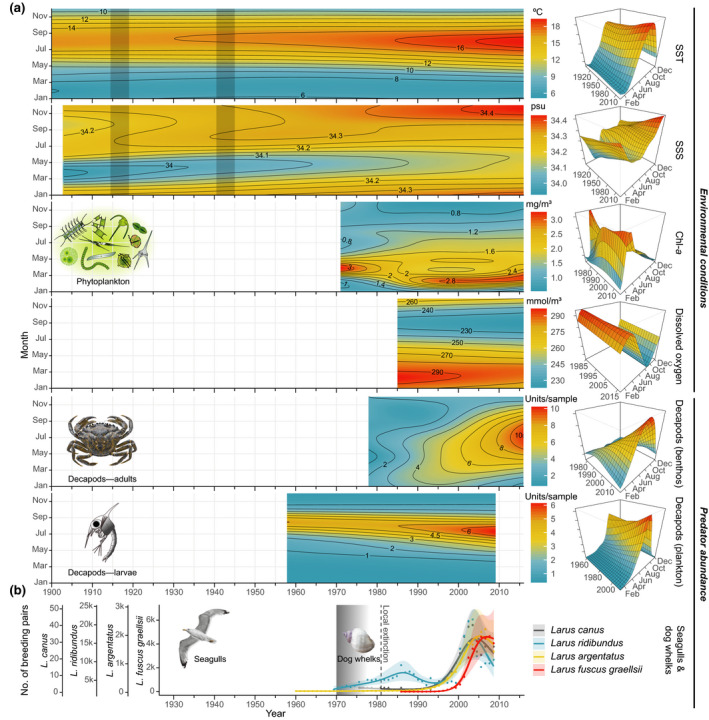
Historical trends and seasonal patterns in local abiotic and biotic conditions over the last century. (a: left panels) Contour plots showing predictions of long‐term changes (between‐year, annual) and variations in the seasonal (within‐year, monthly) patterns of key abiotic and biotic descriptors over the last 116 years along the Belgian study area. Variations in response are represented through colour scales (red: high values; blue: low values) and isolines. Shaded areas for sea surface temperature (SST) and sea surface salinity (SSS) represent historical periods with missing data (1914–1918 and 1940–1945) for which we extrapolated our predictions. (Right panels) Perspective plots of the prediction plane resulting from interactions between long‐term trend and seasonal components of the data. Note the different scales of the ordinate (response, marks as in the colour scale) and abscissa (year axis), highlighting differences in the magnitudes of change and temporal availability for each parameter. (b) Long‐term changes in numbers of breeding pairs in four seagull species. Note the marked among‐species differences in abundance. Simultaneous 95% CIs (shaded areas) and periods of significant change (solid line) are reported. Progressive decrease in *Nucella lapillus* (blended area) during the 1970s and complete disappearance in 1981 (vertical dashed line) from the study location

We identified different temporal patterns depending on the environmental descriptor considered (Figure 3a; Figure S6; Table 1; Table S5). SST significantly increased by 1.10°C between 1975 and 2016 (*F*_2.9,1,064_ = 17.39, *p* < .0001), with a progressively earlier seasonal peak and warmer springs and summers over time (*F*_39.8,1,064_ = 0.97, *p* < .0001; Figure S7). We observed no long‐term variation in annual (*F*_1,1,069_ = 1.19, *p* = .28) and seasonal SSS patterns (*F*_8,1,069_ = 0.05, *p* = .12; Figure S7) between 1903 and 2016. The mean annual Chl‐*a* concentration significantly increased by 0.17 mg/m^3^ between 1971 and 2016 (*F*_1,386_ = 11.11, *p* = .00094). The seasonal Chl‐*a* concentration peaks gradually shifted from spring and summer to winter and spring, with formation of a unique and earlier late‐winter peak after 1990 (*F*_28.6,386_ = 0.98, *p* < .0001; Figure S8). Dissolved oxygen concentration decreased by 6.64 mmol/m^3^ between 1985 and 2016 (*F*_1,374_ = 12.9, *p* = .0004), with no change in seasonality (*F*_0.1,374_ = 0.01, *p* = .72; Figure S8).

**TABLE 1:** GAMMs summary statistics of environmental descriptors and predator abundance. *F*‐values and the significance (n.s., *p* > .01; *, *p* < .01; **, *p* < .001; ***, *p* < .0001) of the trend term (*f*[Year], long‐term change in the mean level of the time series), the seasonal pattern (*f*[Month], within‐year variation), and the interaction between trend and seasonal features of the data (*f*[Year × Month], smooth tensor product interaction) are reported

Parameter	Time range	*n*	Long‐term % change	Trend	Seasonal	Trend × Seasonal
Environment						
SST	1900–2016	1,117	+14.2%	17.39***	894.05***	0.98***
SSS	1903–2016	1,084	+0.31%	1.19^n.s.^	5.35***	0.05^n.s.^
Chl‐*a*	1971–2016	425	+13%	11.11^a^,**	143.24***	0.89***
Dissolved oxygen	1985–2016	384	−2.54%	12.90^a^,**	225.6***	0.01^n.s.^
Decapods						
Benthos	1978–2017	208	+152%	19.87***	1.33**	0.12*
Plankton	1958–2009	624	+92%	12.54^a^,**	92.46***	0.12**
Gulls						
*Larus ridibundus*	1969–2007	39	+258%	11.74***	—	—
*Larus fuscus graellsii*	1985–2007	23	+221k%	399.31***	—	—
*Larus argentatus*	1960–2007	48	+1,658%	624.81***	—	—
*Larus canus*	1975–2007	33	+1,043%	75.06***	—	—

Abbreviations: Chl‐*a*, chlorophyll‐*a*; GAMM, generalized additive mixed‐model; SSS, sea surface salinity; SST, sea surface temperature.

^a^ Linear effect of the covariate on the response.

### 3.3 Historical change of predator abundance

The abundance of macrobenthic decapods (1978–2017) increased 2.5 times (*F*_2.4,197_ = 19.87, *p* < .0001) between spring and autumn (*F*_4.2,197_ = 0.12, *p* = .0072). The abundance of planktonic larvae of decapods (1958–2009) showed a 1.9‐fold increase (*F*_1,605_ = 12.54, *p* = .00043) with the seasonal abundance peak shifting from late to early summer after 1988 (*F*_9.5,605_ = 0.12, *p* = .0007; Figure 3a; Figure S9). Numbers of breeding pairs of the four dominant Belgian seagull species, *L. ridibundus* (black‐headed), *L. canus* (common), *L. argentatus* (European herring), and *L. fuscus graellsii* (lesser black‐backed), increased up to 200–1,500 times between 1960 and 2007 (Figure 3b; Table 1; Table S6). The keystone predatory dog whelk *N. lapillus* began to decrease in numbers after the 1970s and disappeared from the Belgian coast in 1981 (Figure 3b; OSPAR, 2009).

### 3.4 Abiotic and biotic influence on biomineralization

**FIGURE 4:**
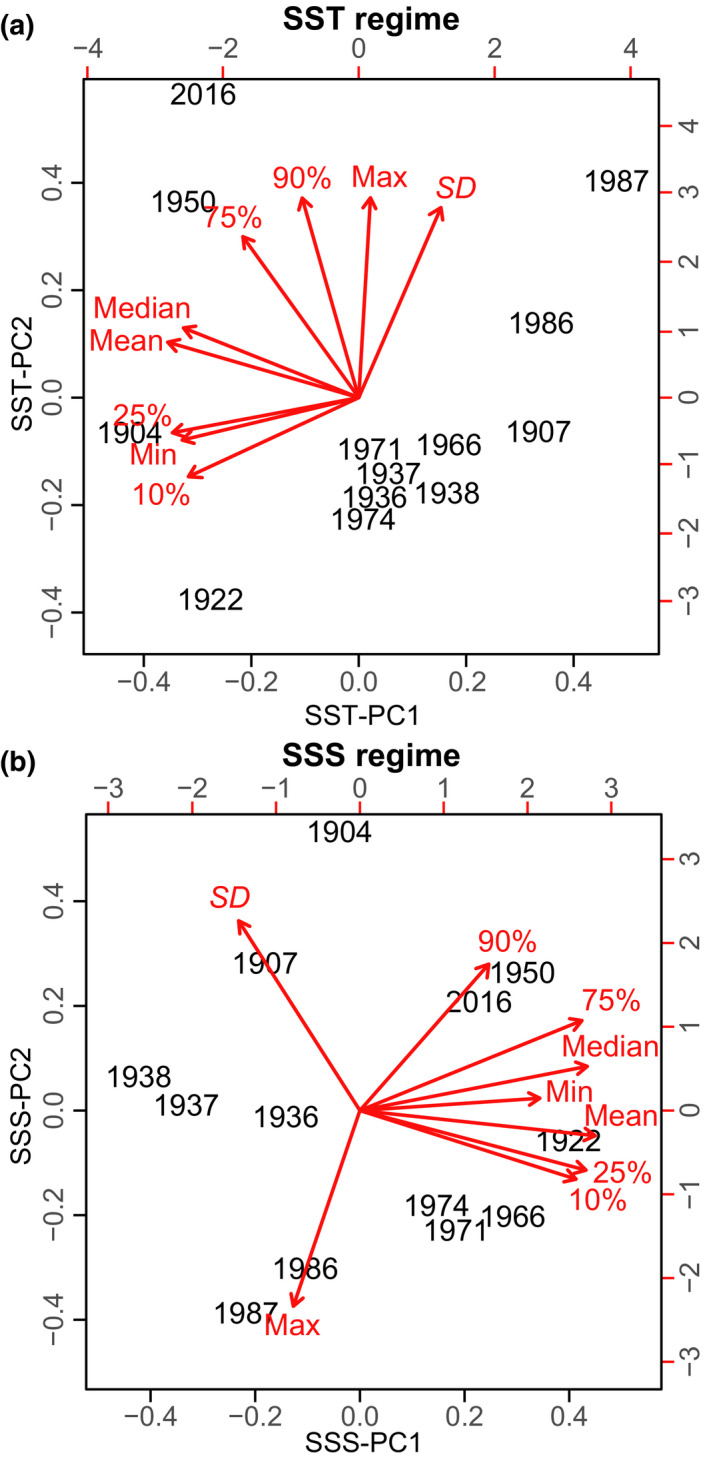
Watertemperature and salinity regimes. Biplots of principal component analyses (PCAs) on (a) sea surface temperature (SST)‐related and (b) sea surface salinity (SSS)‐related descriptors. PCA were performed to (i) create a new set of independent variables and (ii) explore whether shell characteristics were more closely related to estimates of mean conditions (PC1) or the variability of these parameters (PC2). Top and right axes report the loading scores. Red arrows indicate the loading vectors of the original variables on the PCs, showing how each environmental descriptor is related to the plotted PCs (Table S3). The direction of the arrows indicates the direction of increase of the variable values. The position of different collection years on the plane is determined by their relative scores on the plotted PCs. %, parameter percentile; Mean/median, annual mean and median; Min/Max, minimum and maximum values; *SD*, standard deviation

Models indicated differential variations of shell traits with SST and SSS regimes (Figure 4; Table S7). Shell deposition was correlated with changing SST (SST‐PC1: *F*_2.9,250_ = 5.32, *p* = .0019; SST‐PC2: *F*_1,250_ = 7.87, *p* = .0054) and Chl‐*a* (shape‐PC2: *F*_1,250_ = 5.29, *p* = .022). Prismatic layer thickness increased under warmer and more variable SST regimes (SST‐PC1: *F*_4.5,249_ = 22.84, *p* < .0001; SST‐PC2: *F*_1,249_ = 31.59, *p* < .0001), while nacre thickness increased with Chl‐*a* (shape‐PC2: *F*_1,248_ = 5.79, *p* = .017), and under variable SST (SST‐PC2: *F*_3.8,248_ = 4.7, *p* = .0021) and SSS regimes (SSS‐PC2: *F*_1,248_ = 6.22, *p* = .013). Periostraca thickened under less variable SST (SST‐PC2: *F*_2.1,247_ = 4.23, *p* = .0084) and SSS regimes (SSS‐PC2: *F*_2.2,247_ = 3.36, *p* = .044; Figure S10). This suggests that temporal trends in shell deposition were generally more correlated with changes in environmental variability than altered mean water conditions.

**FIGURE 5:**
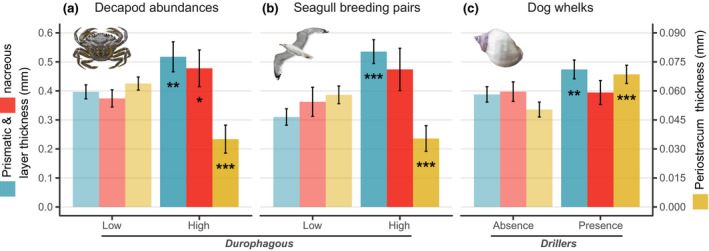
Temporal responses of blue mussel shell biomineralization to changes in predator pressure and strategy. Deposition of individual shell layers (prismatic, blue; nacreous, red; periostracum, yellow) under changing abundance of durophagous and drilling predators. (a) Variations under low (1970–1988) and high (1989–2016) decapod abundances (*n* = 117 × layer) indicating deposition of thicker calcareous layers, and thinner periostraca under high decapod abundances. (b) Shell deposition before (1980–1990) and after (1991–2016) the exponential increase in the number of gull breeding pairs (*n* = 70 × layer) showing thicker prismatic layers and thinner periostraca under increased gull foraging. (c) Composition under presence and absence (before and after 1981, respectively) of *Nucella* *lapillus* (*n* = 117 × layer) showing decreased thickness of prismatic layers and periostraca after the disappearance of dog whelks. Note the dual*y*‐axis highlighting scale differences between shell layers. Error bars indicate 95% CIs (*p < .01; **p < .001; ***p < .0001)

We identified variations in the thickness of each shell layer under different predation types (durophagous vs. drillers) and relative/recorded abundances (Figure 5; Table S8). We observed a 30% increase in deposition of calcareous layers (prismatic: *t*_342_ = 3.94, *p* = .0001; nacre: *t*_342_ = 6.68, *p* = .0061) and formation of 45% thinner periostraca (*t*_342_ = 6.68, *p* < .0001) with higher decapod abundances. Mussels were characterized by 73% thicker prismatic layers (*t*_201_ = 7.93, *p* < .0001) and 39% thinner periostraca (*t*_201_ = −4.92, *p* < .0001) after the exponential increase of seagull breeding pairs between 1991 and 2016. While 22% thinner prismatic layers (*t*_342_ = −4.0, *p* = .0001) and 36% thinner periostraca (*t*_342_ = −5.82, *p* < .0001) were identified after the disappearance of the keystone driller *N. lapillus*.

## 4 DISCUSSION

Although the Belgian coast has been profoundly shaped by rapid urbanization and anthropogenic pressure (Speybroeck et al., 2008), its regular series of stone breakwaters has been preserved almost unchanged over the last 140 years. This breakwater system represents an exceptional long‐term field ‘experimental’ setting for hard‐bottom communities (Warmoes et al., 1988), by providing naturally controlled among‐site conditions in terms of substratum and exposure to changing predation regime, environmental conditions, hydrological, and sedimentary coastal dynamics. The use of a unique long‐term series collection of archival *M. edulis* specimens sampled quasi‐decadally over the last 112 years from geographically well‐delimited Belgian breakwaters, that closely mimic natural hard rock environments, allowed us to identify unexpected natural responses of shell deposition and to evaluate the differential weighting of marked environmental (physicochemical conditions) and ecological (predators abundance) changes on temporal biomineralization patterns. Ecological impacts via rapid changes in predator communities had a stronger influence than long‐term environmental variations on observed compensatory responses in adult mussels that would be expected to follow climate changes according to experimental predictive models.

### 4.1 Historical biomineralization patterns

Contrary to predictions for *Mytilus* spp. and other calcifying foundation species (Gazeau et al., 2013; Pfister et al., 2016), we observed a marked increase in shell deposition and calcification over time. Previous historical studies have indicated shells of modern *Mytilus californianus* (2009–2011) to be 42% thinner than shells from Native American middens (~1,000–2,400 years BP) (Pfister et al., 2016). *Mytilus* larvae and adults produced thinner, weaker, and smaller shells under laboratory‐simulated carbonate chemistries predicted for 2100 (Fitzer et al., 2015; Gaylord et al., 2011). In coralline algae, thallus thickness decreased by 2–2.3 times between 1981 and 2012 showing trade‐offs between growth and calcification (McCoy & Ragazzola, 2014). A 9%–11.4% decline in calcification from 1990 to 2005 has been observed for multiple colonies of the reef‐building coral *Porites* from the Great Barrier Reef (De'ath et al., 2009). However, shell density was reported to increase by 3.4% in the brachiopod *Calloria inconspicua* from New Zealand between 1900 and 2014 (Cross et al., 2018). Differential susceptibilities between species suggest that differences in the potential for compensatory mechanisms, as well as local environmental and ecological alterations, likely govern the wide range of responses among calcifiers that do not always follow global‐level projections.

Laboratory observations have reported shape alterations in *M. edulis* grown in warmer and more acidic waters as a potential adjustment enhancing protection against predators due to their inability to produce thicker shells under physiological stress (Fitzer et al., 2015). The observed lack of clear trends in shell shape but an increase in shell deposition and calcification in a natural system supports the potential of altered species interactions in stimulating changes opposite to what would be predicted under controlled laboratory conditions. This suggests a protective capacity of mussels for structural shell adjustments as compensatory responses to altered environments and predator communities, that are probably entrained over longer timescales from phenotypic (including developmental) plasticity and genetic change in populations via selection.

### 4.2 Long‐term environmental patterns

Our models indicated formation of more variable surface regimes along the Belgian coast over the last century, with temporal patterns depending on the descriptor considered (Figure 3a; Figure S6; Table 1; Table S5). Long‐term increases of SST with warmer spring and summer temperatures are in line with global ocean trends (Desmit et al., 2020; Kirtman et al., 2013). No change in long‐term and seasonal SSS patterns was detected, reflecting expected long‐term stability in local water cycles for areas with intermediate salinity (mid‐latitudes; Kirtman et al., 2013). Increasing Chl‐*a* concentration along the Belgian coast has been widely correlated with growing human activity and land use over the last 60 years, enhancing inorganic nutrients and organic carbon river loads (Desmit et al., 2020; Gypens et al., 2009). These processes have led to increased coastal eutrophication that boosted primary production (Borges & Gypens, 2010; Gypens et al., 2009). The observed changes in timing and onset of Chl‐*a* peaks, with formation of an earlier annual peak in the last two decades is consistent with Desmit et al. (2020). Lower dissolved oxygen concentrations over time are in line with higher SST (decreased oxygen solubility) and primary production, overall increasing biological activity (Figure 3a).

**FIGURE 6:**
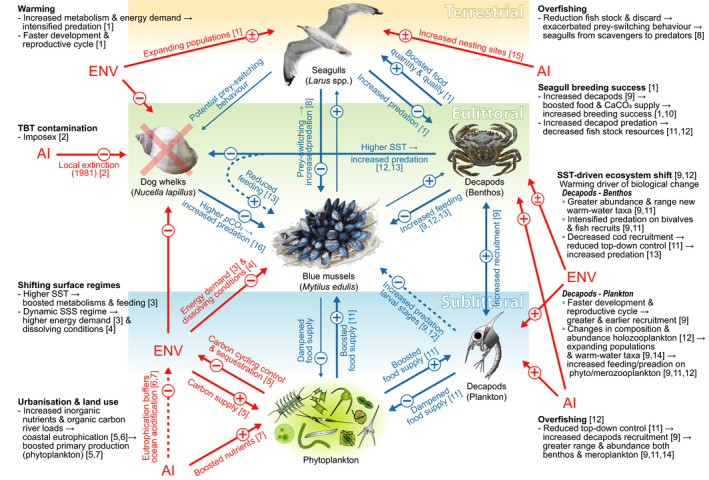
Inferred historical evolution of the blue mussel ecological network. Conceptual representation of end‐to‐end ecosystem interactions (considered in the current study) governing blue mussels beds along the Belgian breakwater system, including primary producers, and primary, secondary, and tertiary consumers from both marine (sublittoral and eulittoral) and terrestrial systems. The graph represents ecological top‐down and bottom‐up control pathways (blue arrows) through which abiotic and biotic interactions within the *Mytilus edulis* ecological network can stimulate compensatory responses of shell biomineralization. Red arrows indicate external environmental (ENV) and anthropogenic (AI) impacts. Signs indicate positive (+) or negative (−) responses. Solid and dashed arrows indicate direct and indirect effects, respectively. Thinner lines indicate likely but speculative interactions. Compensatory adjustments of blue mussel shells represent the shifting balance between responses to propagating climatic and anthropogenic impacts through the durophagous linkages (increasing decapod larvae → decapod adults → seagulls), the disappearance of drillers (dog whelks), and energetically more demanding environmental regimes (increasing sea surface temperature [SST] and fluctuating sea surface salinity [SSS]) in an increasingly productive coastal system (boosted primary productivity and chlorophyll‐*a*); and their chain of direct and indirect feedbacks straightened or weakened by climatic‐ and anthropogenic‐driven stressors. References: [1] (Luczak et al., 2012), [2] (OSPAR, 2009), [3] (Bayne, 1976), [4] (Fitzer et al., 2015), [5] (Gypens et al., 2009), [6] (Borges & Gypens, 2010), [7] (Desmit et al., 2020), [8] (Votier et al., 2004), [9] (Lindley et al., 2010), [10] (Schwemmer & Garthe, 2005), [11] (Kirby et al., 2009), [12] (Kirby & Beaugrand, 2009), [13] (Lord et al., 2017), [14] (Lindley & Kirby, 2010), [15] (Speybroeck et al., 2008), [16] (Sadler et al., 2018)

Our results suggest that formation of more variable sea surface regimes of SST over time has produced energetically more demanding conditions (e.g. from increased basal metabolic costs at higher temperatures) for intertidal mussels (Bayne, 1976; Thomsen et al., 2013; Watson et al., 2017). Local‐scale alterations may, however, benefit indirectly calcification via increased food supply (higher Chl‐*a* concentration) and buffered ocean acidification (Connell et al., 2017; Thomsen et al., 2013; Figure 6). From an historical perspective, observed shell changes, with no evident alteration of shell growth, suggest current mussel populations may have acclimated or adapted to altered environmental conditions along the Belgian coast.

### 4.3 Historical change of predation regime

Our results indicate increasing predation pressures on *M. edulis* with a rapid shift to shell‐breakers (durophagy) dominated regimes (i.e. decapods and seagulls) from the early 1990s on that followed a decline in predation from the local extinction of a keystone predatory driller (dog whelks) at the end of the 1970s (Figures 5 and 6; Table 1).

Long‐term increases in abundance and range of decapods in the North Sea (Lindley et al., 2010; Lindley & Kirby, 2010) have been explained by a temperature‐driven, abrupt ecosystem shift during the 1980s (Kirby & Beaugrand, 2009; Lindley et al., 2010). These changes reflect increasing decapod predation on fish recruits and benthic bivalves (Lindley et al., 2010; Lord et al., 2017), that have both decreased in numbers in the 1990s and 2000s (Kirby et al., 2009; Figure 6). Increasing decapod larvae and shifting seasonal peaks suggest an increased and earlier recruitment (Lindley et al., 2010) that could be favoured by boosted primary production and decreasing top‐down predation pressure due to overfishing and reduced cod recruitment between 1980 and 2009 (Kirby & Beaugrand, 2009; Kirby et al., 2009), decimating commercial fish stocks in the North Sea (Kirby et al., 2009; Votier et al., 2004). Increasing decapod abundances and their potential to amplify effects of climate change at different trophic (Kirby & Beaugrand, 2009) and organizational levels (Luczak et al., 2012) suggest a strong influence of these key predators on the coastal trophic linkages of blue mussels (Lord et al., 2017; Figure 6).

The exponential population growth of seagulls in the 1990s (Figure 3b) has been linked to a trophic amplification of the SST signal through decapods, boosting food supplies for North Sea seagull colonies (Luczak et al., 2012; Figure 6). Seabird breeding success is significantly controlled by prey abundance and composition (Schwemmer & Garthe, 2005), with decapods, pelagic fishes, and fishing discard being dominant components (Votier et al., 2004). A greater abundance of decapods (Kirby et al., 2009; Lindley et al., 2010) may, therefore, benefit seagulls (Luczak et al., 2012) and especially chick development during the breeding season (May–June; Schwemmer & Garthe, 2005), which coincides with the decapod peak abundance (Figure 3). The North Sea supports important fisheries, generating substantial bycatch and discard for seabirds. However, food depletion due to declining volumes of discard (Votier et al., 2004), reduced fish recruitment (Kirby & Beaugrand, 2009), and overfishing (Kirby et al., 2009), has been reported to drive prey‐switching tendencies in generalist scavenging birds, such as seagulls, increasing predation on coastal taxa including mytilids (Votier et al., 2004).

Seagulls have the potential to remove up to 70% of natural and cultured mussel stocks (Nehls et al., 1997). Hence, the population increase in Belgian seagulls (Luczak et al., 2012), coupled with enhanced prey‐switching behaviour (Votier et al., 2004), are key factors boosting predation on coastal resources, among which are the dominant, easily accessible intertidal mussel beds. This illustrates how climate and anthropogenic‐driven marine changes can extend from the avian fauna and propagate to intertidal and terrestrial food webs around seabird colonies (Luczak et al., 2012; Votier et al., 2004; Figure 6).

European populations of *N. lapillus* started declining with the widespread employment of tributyltin in the 1970s, leading to the eradication of the dog whelk from Belgium in 1981 (OSPAR, 2009) and its reappearance along the easternmost part of the Belgian coast (Zeebrugge area) in 2012 (Figure 3b). Dog whelks are key predatory drillers of mussels with a projected stronger selective impact on *M. edulis* under future ocean acidification scenarios (Sadler et al., 2018). Thus, their rapid disappearance would have changed significantly predator–prey coastal dynamics (Figure 6), alleviating predation pressure on mussels.

### 4.4 Triggers of shell compensation

Over the last century, human impact was a major driver of Belgian coastal dynamics with an end‐to‐end ecosystem effect, acting through direct and indirect pathways on the physical–chemical environment, as well as across trophic levels and species interactions governing the blue mussels' ecological network (Figure 6). A limited influence of environmental water regimes on shell deposition (Figure 4) suggest ecological alterations of predator communities in a highly productive system were the principal triggers of the observed responses in biomineralization.

Water temperature is a key factor in controlling growth and physiological activity in marine calcifiers (Bayne, 1976; Gazeau et al., 2013). Previous research demonstrated rising temperatures within a species ecological range can increase calcification in bivalves (Kroeker et al., 2014; Telesca et al., 2019), with *M. edulis* showing optimal growth between 10°C and 20°C (Bayne, 1976). Studies on *M. galloprovincialis* grown under acidified (variable seawater CO_2_ partial pressure, or *p*CO_2_) and warmer waters have reported an average +2.63% and +2% increase in shell deposition per unit size for a 1°C increase under low (~400 μatm) and high (~1,200 μatm) *p*CO_2_ conditions, respectively (Kroeker et al., 2014). Moreover, a large‐scale study (Telesca et al., 2019) predicts an increase of +3.3% in both prismatic and nacreous layer thicknesses for a 1°C increase under SSS and Chl‐*a* conditions recorded along the Belgian coastline. Although increases in average annual (+1°C) and summer (+2.7°C) SSTs along the Belgian coast may have contributed positively to shell calcification, temperature patterns alone explain only a small proportion of historical increases in biomineralization (a total of +57% between 1904 and 2016).

Food supply is potentially the most important factor supporting growth in mussels (Bayne, 1976; Thomsen et al., 2013), but this strongly covaries with water temperature. Increased food supply (Chl‐*a*) supports *Mytilus* calcification (Thomsen et al., 2013), and increased organic content in shells (Telesca et al., 2019) and allows sustained growth over a wide range of environmental temperatures (5°C–20°C; Bayne, 1976). Although increased Chl‐*a* concentrations in response to eutrophication processes along the Belgian coast (Figure 3a) could be beneficial to mussels (Thomsen et al., 2013), the positive correlation between food supply and shell growth decreases with increasing mussel size, and has no effect on adults larger than 40–50 mm (Page & Hubbard, 1987) that represent the lower tail of the shell size classes included in our study. This correlation is also supported by Telesca et al. (2019) who reported no relationship between Chl‐*a* levels and the deposition of calcareous shell components in adult mussels (>35 mm) across latitudes.

Bivalve calcification is particularly sensitive to changes of marine carbonate chemistry resulting from the increased absorption of atmospheric CO_2_ by the ocean. CO_2_ uptake increases the dissolved inorganic carbon (DIC), in particular seawater *p*CO_2_ and ${\text{HCO}}_{3}^{‐}$, controlling both pH and the thermodynamic mineral solubility (saturation state). Increasing *p*CO_2_ decreases seawater $\left[{\text{CO}}_{3}^{2‐}\right]$ and pH, causing a corresponding decrease in CaCO_3_ saturation state and acidification of the oceans, that interfere with calcification in marine organisms (Gazeau et al., 2013; Thomsen et al., 2015; Waldbusser et al., 2015). Changes in $\left[{\text{CO}}_{3}^{2‐}\right]$ and, therefore, saturation state impact bivalve calcification directly, through dissolution or by preventing early shell formation (Thomsen et al., 2015; Waldbusser et al., 2015). Differently, reductions in seawater pH and increased *p*CO_2_ impact calcification indirectly via disturbance of physiological processes and reductions in scope for growth (Thomsen et al., 2015). Under future climate scenarios changes in marine carbonate chemistry are predicted to have deleterious effects on calcification in bivalves and their ecological functions (Gazeau et al., 2013; Waldbusser et al., 2015).

In the Belgian coastal zone, Desmit et al. (2020) reported a peak of pH values between 1975 and 1988, followed by an overall long‐term stability from 1989 to 2015 and progressively more marked seasonal changes over time, that corroborates the observed shift of seasonal Chl‐*a* concentration peaks (Figure 3a; Figure S8). Reported pH trends are in line with the strong impact of altered phytoplankton communities on coastal carbon cycling and sequestration (Gypens et al., 2009), as well as eutrophication and increased riverine nutrient inputs (increased alkalinity) buffering ocean acidification in surface coastal waters (Borges & Gypens, 2010). This pattern supports how the coastal zones will not experience acidification gradually, as in the open oceans, but rather as increased frequency, duration, and magnitude of unfavourable events for coastal ecologically and economically valuable species (Waldbusser et al., 2015). However, reported pH changes may reflect nonlinear alterations in carbonate parameters (i.e. *p*CO_2_, saturation state, DIC) that cannot be easily reconstructed for our study location. This covariation of carbonate system variables indicates the importance of complete carbonate chemistry monitoring and the potential limitations of historical datasets, which are mainly focused on pH records, in linking calcifiers' biology to water chemistry observations.

Historical alterations in other local processes due to the rapid urban development of the Belgian coast (Speybroeck et al., 2008), which could not be directly included in our analysis, such as changes in riverine input and sediment transport, might have influenced small‐scale coastal gradients with potential (in)direct effects on mussel beds. Most freshwater inputs are characterized by lower pH than oceanic waters, mainly due to low alkalinity and high *p*CO_2_ originating from higher loads of organic matter (Desmit et al., 2020). Historical trends of riverine inputs in the southern North Sea regions indicate decreasing volumes of freshwater discharge after the 1980s (van Beusekom et al., 2019). Reductions of river runoffs might lead to alterations of cross‐shore SSS gradients across short distances (higher salinity), increasing the saturation state of calcium carbonate (Thomsen et al., 2015), with the potential formation of localized areas along the Belgian coast with more suitable environment for mussel calcification (Telesca et al., 2019). Moreover, long‐term increases in dune volume have been reported along most of the Belgian coastline after the 1980s (Strypsteen et al., 2019). However, changes in associated sediment volume and transport might have had only indirect implications for shell biomineralization through physical influences on mussel beds dynamics.

*Mytilus edulis* fitness is tightly linked to shell integrity, deposition, and composition (Telesca et al., 2019), and to its capacity to change shell production in response to the presence or absence of predators (Freeman, 2007; Lowen et al., 2013; Sherker et al., 2017). The organic periostracum provides protection against predatory and endolithic borers as well as corrosive waters (Harper, 1997; Harper & Skelton, 1993). Increased deposition of calcareous layers improves defences in thicker‐shelled individuals against durophagous and drilling predators (Freeman, 2007; Sherker et al., 2017). Given the differential defensive value of shell layers, differences in energetic costs of CaCO_3_ (low) versus organics (high) production (Watson et al., 2017), as well as marked environmental and ecological alterations, mussels will be under strong selection pressure favouring the capacity to modify shell production to cope with a range of disturbances.

Although reported environmental changes could have a positive but relatively minor contribution on *Mytilus* calcification, temporal changes in predation pressure and strategy were the key drivers of the observed historical increase in shell deposition (Figures 1c and 5). Shell thickening and increased calcification are in line with increasing decapod abundances and the inducible defence responses to crab predation (Freeman, 2007; Lowen et al., 2013; Figure 5). Prismatic layer thickness has been documented as the factor largely determining *M. edulis* shell vulnerability to seabird predation (Le Rossignol et al., 2011). This suggests the rapid prismatic layer thickening after 1995 (Figure 5) likely reflects a response to the exponential growth of seagull colonies (Figures 3b and 6). Similarly, the rapid periostracal thinning (−29%) after 1984 and less organic matrix (−13%) in modern shells (Figures 2a and 5) suggest compensatory responses to the disappearance of *N. lapillus* (1981) and to buffered ocean acidification effects (Borges & Gypens, 2010), both selecting for shells with higher resistance to dissolution (i.e. thick periostracum and abundant organic matrix; Harper & Skelton, 1993; Telesca et al., 2019; Thomsen et al., 2017). Although past shell patterns cannot be fully explained due to the limited historical records, our findings support the potential of mussels for adjustments in the deposition of calcareous and organic shell components as a compensatory response to predation strategy‐specific pressures.

Our results highlight how complex, local‐scale changes in ecological conditions can trigger compensatory mechanisms and lead to unanticipated biological outcomes that run contrary to global‐scale predictions. In this regional scenario (Figure 6), the disappearance of a keystone predator, selecting for energetically ‘expensive’ responses (periostracum‐ and organic‐enriched shells), increased food supply via eutrophication, and buffered ocean acidification, likely led to compositional trade‐offs with formation of ‘cheaper’, calcareous‐dominated (decreased organic content) shells as a response to increased durophagous predation.

### 4.5 Archival collections as key tools for environmental change research

The use of archival specimens collected regularly from geographically well‐delimited locations over the last century (1904–2016) allowed the characterization of overall temporal responses, incorporating combined impacts of long‐term acclimation and adaption, in a calcifying foundation species within a system that closely mimics intertidal hard rock environments (Figure 6).

Our findings demonstrate a surprising capacity of *Mytilus* to withstand a wide range of environmental perturbations and altered predation impacts through unexpected levels of qualitative and quantitative compensatory adjustments in shell biomineralization. Increasing evidence supports the potential for resistance in calcifiers to environmental changes (Cross et al., 2018, 2019; Telesca et al., 2019; Thomsen et al., 2017), suggesting compensatory biomineralization to disturbance may act in a range of organisms, being driven by species‐specific skeletal structures, physiological plasticity, and genetic adaption. This highlights its potential value in calcifiers to maintain their ecological functions in rapidly changing environments via formation of skeletal structures reflecting the interactions between climatic, ecological, and anthropogenic selective forces (Figure 6), and the cost of compensation.

Our results also highlight the potential role of local ecological changes of predator communities in stimulating unexpected compensatory responses of biomineralization in adult mussels that we often assume will be less accentuated than in early life‐history stages and follow environmental change according to experimental predictive models. Altered species interactions represent a key leverage point through which compensatory mechanisms could indirectly attenuate the direct impacts of rapidly changing climates on natural populations. As climate change accelerates, local anthropogenic impacts may determine future shifts in the balance of environmental and ecological forces on calcifiers and lead to unanticipated biological patterns and ecosystem alterations.

Compensatory biomineralization represents a potentially important, but still poorly explored mechanism for resistance in calcifiers to ecological and environmental change complementing other physiological, population, and community‐level adjustments (Kroeker et al., 2017; Peck et al., 2015). A better understanding of the mechanisms driving biological variation is needed to inform more realistic projections of organismal responses to climate and anthropogenic impacts (Vargas et al., 2017), and to guide more effective environmental management policies (Urban et al., 2016). In this regard, historical studies of archival records represent a rarely used but powerful approach that, as here, can identify unexpected responses to change and also complement experimental approaches to provide a more integrative assessment of the direct and indirect pathways of ecological change or stability.

## AUTHOR CONTRIBUTION

L.T., L.S.P., T.B., and E.M.H. conceived the original project and designed the study; L.T. performed the specimen collection, laboratory work, generated environmental datasets, performed modelling work, and analysed output data; M.H. performed the thermogravimetric analysis; T.B. provided the archival specimens; L.T. collected the living specimens; L.T., L.S.P., and E.M.H. wrote the first draft of the manuscript, and all co‐authors contributed substantially to revisions.

## Supporting information

Supplementary Material

## Data Availability

Datasets generated during this study are made available through the Figshare data repository and can be accessed at https://doi.org/10.6084/m9.figshare.13125293.

## References

[gcb15417-bib-0001] Ashton, G. V. , Morley, S. A. , Barnes, D. K. A. , Clark, M. S. , & Peck, L. S. (2017). Warming by 1°C drives species and assemblage level responses in Antarctica's marine shallows. Current Biology, 27(17), 2698–2705.e3. 10.1016/j.cub.2017.07.048 28867203

[gcb15417-bib-0002] Bayne, B. L. (1976). Marine mussels: Their ecology and physiology. Cambridge University Press.

[gcb15417-bib-0003] Bolker, B. M. (2015). Linear and generalized linear mixed models In Fox G. A., Negrete‐Yankelevich S., & Sosa V. J. (Eds.), Ecological statistics (pp. 309–333). Oxford University Press 10.1093/acprof:oso/9780199672547.003.0014

[gcb15417-bib-0004] Borges, A. V. , & Gypens, N. (2010). Carbonate chemistry in the coastal zone responds more strongly to eutrophication than ocean acidification. Limnology and Oceanography, 55(1), 346–353. 10.4319/lo.2010.55.1.0346

[gcb15417-bib-0005] Clark, M. S. , Villota Nieva, L. , Hoffman, J. I. , Davies, A. J. , Trivedi, U. H. , Turner, F. , Ashton, G. V. , & Peck, L. S. (2019). Lack of long‐term acclimation in Antarctic encrusting species suggests vulnerability to warming. Nature Communications, 10(1), 3383 10.1038/s41467-019-11348-w PMC6662708 31358752

[gcb15417-bib-0006] Connell, S. D. , Doubleday, Z. A. , Hamlyn, S. B. , Foster, N. R. , Harley, C. D. G. , Helmuth, B. , Kelaher, B. P. , Nagelkerken, I. , Sarà, G. , & Russell, B. D. (2017). How ocean acidification can benefit calcifiers. Current Biology, 27(3), R95–R96. 10.1016/j.cub.2016.12.004 28171763

[gcb15417-bib-0007] Cross, E. L. , Harper, E. M. , & Peck, L. S. (2018). A 120‐year record of resilience to environmental change in brachiopods. Global Change Biology, 24(6), 2262–2271. 10.1111/gcb.14085 29536586 PMC6850138

[gcb15417-bib-0008] Cross, E. L. , Harper, E. M. , & Peck, L. S. (2019). Thicker shells compensate extensive dissolution in brachiopods under future ocean acidification. Environmental Science & Technology, 53(9), 5016–5026. 10.1021/acs.est.9b00714 30925214

[gcb15417-bib-0009] De'ath, G. , Lough, J. M. , & Fabricius, K. E. (2009). Declining coral calcification on the Great Barrier Reef. Science, 323(5910), 116–119. 10.1126/science.1165283 19119230

[gcb15417-bib-0010] Desmit, X. , Nohe, A. , Borges, A. V. , Prins, T. , De Cauwer, K. , Lagring, R. , Van der Zande, D. , & Sabbe, K. (2020). Changes in chlorophyll concentration and phenology in the North Sea in relation to de‐eutrophication and sea surface warming. Limnology and Oceanography, 65(4), 828–847. 10.1002/lno.11351

[gcb15417-bib-0011] Fitzer, S. C. , Vittert, L. , Bowman, A. , Kamenos, N. A. , Phoenix, V. R. , & Cusack, M. (2015). Ocean acidification and temperature increase impact mussel shell shape and thickness: Problematic for protection? Ecology and Evolution, 5(21), 4875–4884. 10.1002/ece3.1756 26640667 PMC4662322

[gcb15417-bib-0012] Freeman, A. S. (2007). Specificity of induced defenses in *Mytilus edulis* and asymmetrical predator deterrence. Marine Ecology Progress Series, 334, 145–153. 10.3354/meps334145

[gcb15417-bib-0013] Gaylord, B. , Hill, T. M. , Sanford, E. , Lenz, E. A. , Jacobs, L. A. , Sato, K. N. , Russell, A. D. , & Hettinger, A. (2011). Functional impacts of ocean acidification in an ecologically critical foundation species. Journal of Experimental Biology, 214(15), 2586–2594. 10.1242/jeb.055939 21753053

[gcb15417-bib-0014] Gazeau, F. , Parker, L. M. , Comeau, S. , Gattuso, J.‐P. , O'Connor, W. A. , Martin, S. , Pörtner, H.‐O. , & Ross, P. M. (2013). Impacts of ocean acidification on marine shelled molluscs. Marine Biology, 160(8), 2207–2245. 10.1007/s00227-013-2219-3

[gcb15417-bib-0015] Ghedini, G. , Russell, B. D. , & Connell, S. D. (2015). Trophic compensation reinforces resistance: Herbivory absorbs the increasing effects of multiple disturbances. Ecology Letters, 18(2), 182–187. 10.1111/ele.12405 25581377

[gcb15417-bib-0016] Gypens, N. , Borges, A. V. , & Llancelot, C. (2009). Effect of eutrophication on air‐sea CO_2_ fluxes in the coastal Southern North Sea: A model study of the past 50 years. Global Change Biology, 15(4), 1040–1056. 10.1111/j.1365-2486.2008.01773.x

[gcb15417-bib-0017] Harper, E. M. (1997). The molluscan periostracum: An important constraint in bivalve evolution. Palaeontology, 40(1), 71–97. Retrieved from http://www.scopus.com/inward/record.url?eid=2‐s2.0‐0030782099&partnerID=40

[gcb15417-bib-0018] Harper, E. M. , & Skelton, P. W. (1993). A defensive value of the thickened periostracum in the Mytiloidea. Veliger, 36, 36–42.

[gcb15417-bib-0019] Kirby, R. R. , & Beaugrand, G. (2009). Trophic amplification of climate warming. Proceedings of the Royal Society B: Biological Sciences, 276(1676), 4095–4103. 10.1098/rspb.2009.1320 PMC2821349 19740882

[gcb15417-bib-0020] Kirby, R. R. , Beaugrand, G. , & Lindley, J. A. (2009). Synergistic effects of climate and fishing in a marine ecosystem. Ecosystems, 12(4), 548–561. 10.1007/s10021-009-9241-9

[gcb15417-bib-0021] Kirtman, B. , Power, S. B. , Adedoyin, J. A. , Boer, G. J. , Bojariu, R. , Camilloni, I. , & Wang, H. J. (2013). Near‐term climate change: Projections and predictability In Stocker T. F., Qin D., Plattner G.‐K., Tignor M., Allen S. K., Boschung J., & Midgley P. M. (Eds.), Climate change 2013: The physical science basis. Contribution of working group I to the fifth assessment report of the Intergovernmental Panel on Climate Change (pp. 953–1028). Cambridge University Press.

[gcb15417-bib-0022] Kroeker, K. J. , Gaylord, B. , Hill, T. M. , Hosfelt, J. D. , Miller, S. H. , & Sanford, E. (2014). The role of temperature in determining species' vulnerability to ocean acidification: A case study using *Mytilus galloprovincialis* . PLoS One, 9(7), e100353 10.1371/journal.pone.0100353 24984016 PMC4077567

[gcb15417-bib-0023] Kroeker, K. J. , Kordas, R. L. , Crim, R. , Hendriks, I. E. , Ramajo, L. , Singh, G. S. , Duarte, C. M. , & Gattuso, J.‐P. (2013). Impacts of ocean acidification on marine organisms: Quantifying sensitivities and interaction with warming. Global Change Biology, 19(6), 1884–1896. 10.1111/gcb.12179 23505245 PMC3664023

[gcb15417-bib-0024] Kroeker, K. J. , Kordas, R. L. , & Harley, C. D. G. (2017). Embracing interactions in ocean acidification research: Confronting multiple stressor scenarios and context dependence. Biology Letters, 13(3), 20160802 10.1098/rsbl.2016.0802 28356409 PMC5377028

[gcb15417-bib-0025] Kroeker, K. J. , Sanford, E. , Rose, J. M. , Blanchette, C. A. , Chan, F. , Chavez, F. P. , Gaylord, B. , Helmuth, B. , Hill, T. M. , Hofmann, G. E. , McManus, M. A. , Menge, B. A. , Nielsen, K. J. , Raimondi, P. T. , Russell, A. D. , & Washburn, L. (2016). Interacting environmental mosaics drive geographic variation in mussel performance and predation vulnerability. Ecology Letters, 19(7), 771–779. 10.1111/ele.12613 27151381

[gcb15417-bib-0026] Le Rossignol, A. P. , Buckingham, S. G. , Lea, S. E. G. , & Nagarajan, R. (2011). Breaking down the mussel (*Mytilus edulis*) shell: Which layers affect Oystercatchers' (*Haematopus ostralegus*) prey selection? Journal of Experimental Marine Biology and Ecology, 405(1–2), 87–92. 10.1016/j.jembe.2011.05.021

[gcb15417-bib-0027] Lindley, J. A. , Beaugrand, G. , Luczak, C. , Dewarumez, J.‐M. , & Kirby, R. R. (2010). Warm‐water decapods and the trophic amplification of climate in the North Sea. Biology Letters, 6(6), 773–776. 10.1098/rsbl.2010.0394 20554562 PMC3001376

[gcb15417-bib-0028] Lindley, J. A. , & Kirby, R. (2010). Climate‐induced changes in the North Sea Decapoda over the last 60 years. Climate Research, 42(3), 257–264. 10.3354/cr00917

[gcb15417-bib-0029] Lord, J. , Barry, J. , & Graves, D. (2017). Impact of climate change on direct and indirect species interactions. Marine Ecology Progress Series, 571, 1–11. 10.3354/meps12148

[gcb15417-bib-0030] Lowen, J. , Innes, D. , & Thompson, R. (2013). Predator‐induced defenses differ between sympatric *Mytilus edulis* and *M. trossulus* . Marine Ecology Progress Series, 475, 135–143. 10.3354/meps10106

[gcb15417-bib-0031] Luczak, C. , Beaugrand, G. , Lindley, J. A. , Dewarumez, J.‐M. , Dubois, P. J. , & Kirby, R. R. (2012). North Sea ecosystem change from swimming crabs to seagulls. Biology Letters, 8(5), 821–824. 10.1098/rsbl.2012.0474 22764111 PMC3441004

[gcb15417-bib-0032] McCoy, S. J. , & Ragazzola, F. (2014). Skeletal trade‐offs in coralline algae in response to ocean acidification. Nature Climate Change, 4(8), 719–723. 10.1038/nclimate2273

[gcb15417-bib-0033] Nagelkerken, I. , & Connell, S. D. (2015). Global alteration of ocean ecosystem functioning due to increasing human CO_2_ emissions. Proceedings of the National Academy of Sciences of the United States of America, 112(43), 13272–13277. 10.1073/pnas.1510856112 26460052 PMC4629388

[gcb15417-bib-0034] Nehls, G. , Hertzler, I. , & Scheiffarth, G. (1997). Stable mussel *Mytilus edulis* beds in the Wadden Sea – They're just for the birds. Helgoländer Meeresuntersuchungen, 51(3), 361–372. 10.1007/BF02908720

[gcb15417-bib-0035] OSPAR . (2009). Background document for *Nucella lapillus* (Dog whelk). Biodiversity Series. OSPAR.

[gcb15417-bib-0036] Page, H. , & Hubbard, D. M. (1987). Temporal and spatial patterns of growth in mussels *Mytilus edulis* on an offshore platform: Relationships to water temperature and food availability. Journal of Experimental Marine Biology and Ecology, 111, 159–179. 10.1016/0022-0981(87)90053-0

[gcb15417-bib-0037] Peck, L. S. , Clark, M. S. , Power, D. , Reis, J. , Batista, F. M. , & Harper, E. M. (2015). Acidification effects on biofouling communities: Winners and losers. Global Change Biology, 21(5), 1907–1913. 10.1111/gcb.12841 25626420 PMC5006883

[gcb15417-bib-0038] Pfister, C. A. , Roy, K. , Wootton, J. T. , McCoy, S. J. , Paine, R. T. , Suchanek, T. H. , & Sanford, E. (2016). Historical baselines and the future of shell calcification for a foundation species in a changing ocean. Proceedings of the Royal Society B: Biological Sciences, 283(1832), 20160392 10.1098/rspb.2016.0392 PMC4920315 27306049

[gcb15417-bib-0039] R Core Team . (2018). R: A language and environment for statistical computing. Retrieved from https://www.r‐project.org/

[gcb15417-bib-0040] Sadler, D. E. , Lemasson, A. J. , & Knights, A. M. (2018). The effects of elevated CO_2_ on shell properties and susceptibility to predation in mussels *Mytilus edulis* . Marine Environmental Research, 139, 162–168. 10.1016/J.MARENVRES.2018.05.017 29803323

[gcb15417-bib-0041] Schwemmer, P. , & Garthe, S. (2005). At‐sea distribution and behaviour of a surface‐feeding seabird, the lesser black‐backed gull *Larus fuscus*, and its association with different prey. Marine Ecology Progress Series, 285, 245–258. 10.3354/meps285245

[gcb15417-bib-0042] Seed, R. (1969). The ecology of *Mytilus edulis* L. (Lamellibranchiata) on exposed rocky shores: II. Growth and mortality. Oecologia, 3(3–4), 317–350. 10.1007/BF00390381 28308906

[gcb15417-bib-0043] Seed, R. , & Suchanek, T. H. (1992). Population and community ecology of *Mytilus* In Gosling E. M. (Ed.), The mussel *Mytilus*: Ecology, physiology, genetics and culture (pp. 87–170). Elsevier.

[gcb15417-bib-0044] Sherker, Z. , Ellrich, J. , & Scrosati, R. (2017). Predator‐induced shell plasticity in mussels hinders predation by drilling snails. Marine Ecology Progress Series, 573, 167–175. 10.3354/meps12194

[gcb15417-bib-0045] Speybroeck, J. , Bonte, D. , Courtens, W. , Gheskiere, T. , Grootaert, P. , Maelfait, J.‐P. , Provoost, S. , Sabbe, K. , Stienen, E. W. M. , Van Lancker, V. , Van Landuyt, W. , Vincx, M. , & Degraer, S. (2008). The Belgian sandy beach ecosystem: A review. Marine Ecology, 29(s1), 171–185. 10.1111/j.1439-0485.2008.00232.x

[gcb15417-bib-0046] Strypsteen, G. , Houthuys, R. , & Rauwoens, P. (2019). Dune volume changes at decadal timescales and its relation with potential aeolian transport. Journal of Marine Science and Engineering, 7(10), 357 10.3390/jmse7100357

[gcb15417-bib-0047] Telesca, L. , Michalek, K. , Sanders, T. , Peck, L. S. , Thyrring, J. , & Harper, E. M. (2018). Blue mussel shell shape plasticity and natural environments: A quantitative approach. Scientific Reports, 8(1), 2865 10.1038/s41598-018-20122-9 29434221 PMC5809382

[gcb15417-bib-0048] Telesca, L. , Peck, L. S. , Sanders, T. , Thyrring, J. , Sejr, M. K. , & Harper, E. M. (2019). Biomineralization plasticity and environmental heterogeneity predict geographical resilience patterns of foundation species to future change. Global Change Biology, 25(12), 4179–4193. 10.1111/gcb.14758 31432587

[gcb15417-bib-0049] Thomsen, J. , Casties, I. , Pansch, C. , Körtzinger, A. , & Melzner, F. (2013). Food availability outweighs ocean acidification effects in juvenile *Mytilus edulis*: Laboratory and field experiments. Global Change Biology, 19(4), 1017–1027. 10.1111/gcb.12109 23504880

[gcb15417-bib-0050] Thomsen, J. , Haynert, K. , Wegner, K. M. , & Melzner, F. (2015). Impact of seawater carbonate chemistry on the calcification of marine bivalves. Biogeosciences, 12(14), 4209–4220. 10.5194/bg-12-4209-2015

[gcb15417-bib-0051] Thomsen, J. , Stapp, L. S. , Haynert, K. , Schade, H. , Danelli, M. , Lannig, G. , Wegner, K. M. , & Melzner, F. (2017). Naturally acidified habitat selects for ocean acidification – Tolerant mussels. Science Advances, 3(4), e1602411 10.1126/sciadv.1602411 28508039 PMC5406135

[gcb15417-bib-0052] Urban, M. C. , Bocedi, G. , Hendry, A. P. , Mihoub, J.‐B. , Peer, G. , Singer, A. , Bridle, J. R. , Crozier, L. G. , De Meester, L. , Godsoe, W. , Gonzalez, A. , Hellmann, J. J. , Holt, R. D. , Huth, A. , Johst, K. , Krug, C. B. , Leadley, P. W. , Palmer, S. C. F. , Pantel, J. H. , … Travis, J. M. J. (2016). Improving the forecast for biodiversity under climate change. Science, 353(6304), aad8466 10.1126/science.aad8466 27609898

[gcb15417-bib-0053] van Beusekom, J. E. E. , Carstensen, J. , Dolch, T. , Grage, A. , Hofmeister, R. , Lenhart, H. , Kerimoglu, O. , Kolbe, K. , Pätsch, J. , Rick, J. , Rönn, L. , & Ruiter, H. (2019). Wadden Sea eutrophication: Long‐term trends and regional differences. Frontiers in Marine Science, 6, 370 10.3389/fmars.2019.00370

[gcb15417-bib-0054] Vargas, C. A. , Lagos, N. A. , Lardies, M. A. , Duarte, C. , Manríquez, P. H. , Aguilera, V. M. , Broitman, B. , Widdicombe, S. , & Dupont, S. (2017). Species‐specific responses to ocean acidification should account for local adaptation and adaptive plasticity. Nature Ecology & Evolution, 1(4), 0084 10.1038/s41559-017-0084 28812677

[gcb15417-bib-0055] Votier, S. C. , Furness, R. W. , Bearhop, S. , Crane, J. E. , Caldow, R. W. G. , Catry, P. , Ensor, K. , Hamer, K. C. , Hudson, A. V. , Kalmbach, E. , Klomp, N. I. , Pfeiffer, S. , Phillips, R. A. , Prieto, I. , & Thompson, D. R. (2004). Changes in fisheries discard rates and seabird communities. Nature, 427(6976), 727–730. 10.1038/nature02315 14973483

[gcb15417-bib-0056] Waldbusser, G. G. , Hales, B. , Langdon, C. J. , Haley, B. A. , Schrader, P. , Brunner, E. L. , Gray, M. W. , Miller, C. A. , & Gimenez, I. (2015). Saturation‐state sensitivity of marine bivalve larvae to ocean acidification. Nature Climate Change, 5(3), 273–280. 10.1038/nclimate2479

[gcb15417-bib-0057] Warmoes, T. , Backeljau, T. , & De Bruyn, L. (1988). The littorinid fauna of the Belgian coast (Mollusca, Gastropoda). Bulletin de L'institut Royal Des Sciences Naturelles de Belgique, Biologie, 58, 51–70.

[gcb15417-bib-0058] Watson, S.‐A. , Morley, S. A. , & Peck, L. S. (2017). Latitudinal trends in shell production cost from the tropics to the poles. Science Advances, 3(9), e1701362 10.1126/sciadv.1701362 28948224 PMC5606708

[gcb15417-bib-0059] Wood, S. N. (2017). Generalized additive models: An introduction with R (2nd ed.). Chapman & Hall/CRC.

[gcb15417-bib-0060] Zuur, A. F. , Ieno, E. N. , & Elphick, C. S. (2010). A protocol for data exploration to avoid common statistical problems. Methods in Ecology and Evolution, 1(1), 3–14. 10.1111/j.2041-210X.2009.00001.x

