# Supplementary material for: A century of coping with environmental and ecological changes via compensatory biomineralization in mussels
Source: Glob Chang Biol. 2020 Nov 21;27(3):624–39. doi: 10.1111/gcb.15417 (PMC7839727; doi:10.1111/gcb.15417)
Supplement: Supplementary file 1 — Supplementary Material [file GCB-27-624-s001.pdf]

Supplementary Information for

A century of coping with environmental and ecological changes *via* compensatory biomineralization in mussels

Luca Telesca, Lloyd S. Peck, Thierry Backeljau, Mario F. Heinig, Elizabeth M. Harper

List of Supplementary Figures

Figure S1 2
Figure S2 3
Figure S3 4
Figure S4 5
Figure S5 6
Figure S6 7
Figure S7 8
Figure S8 9
Figure S9 10
Figure S10 11

List of Supplementary Tables

Table S1 12
Table S2 13
Table S3 14
Table S4 15
Table S5 16
Table S6 17
Table S7 18
Table S8 19

Supplementary Documents

Supplementary Methods 20
Supplementary Data 25

References **29**

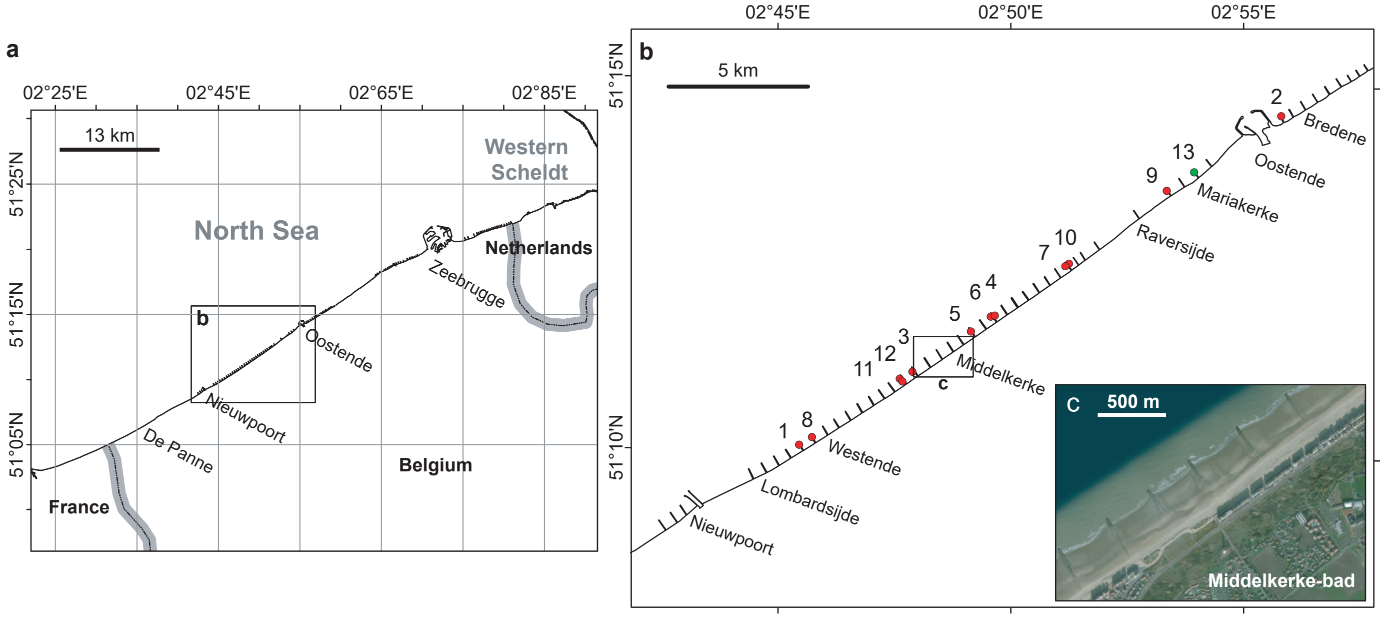

Figure S1 ***Mytilus edulis*** **collection sites: the Belgian coast and its breakwaters system. (a)** The Belgian coastal system and collection area stretching from Calais in France to the Westerschelde estuary in the Netherlands. **(b)** Map of the sampling locations between Oostende and Nieuwpoort (51°09'14.14"N - 51°14'16.27"N and 2°43'23.6"E - 2°55'03.0"E) showing the sites where archival (1-12, red circles) and modern (13, green circle) specimens of *Mytilus edulis* were collected. **(c)** Detail of the regular series of stone breakwaters (during low-tide) characterising the coastline of the sampling location (see Supplementary Methods). Maps created with ArcMap 10.5 (ArcGIS software by Esri, `http://esri.com`), background images and topographic details courtesy of OpenStreetMap (`http://www.openstreetmap.org`).

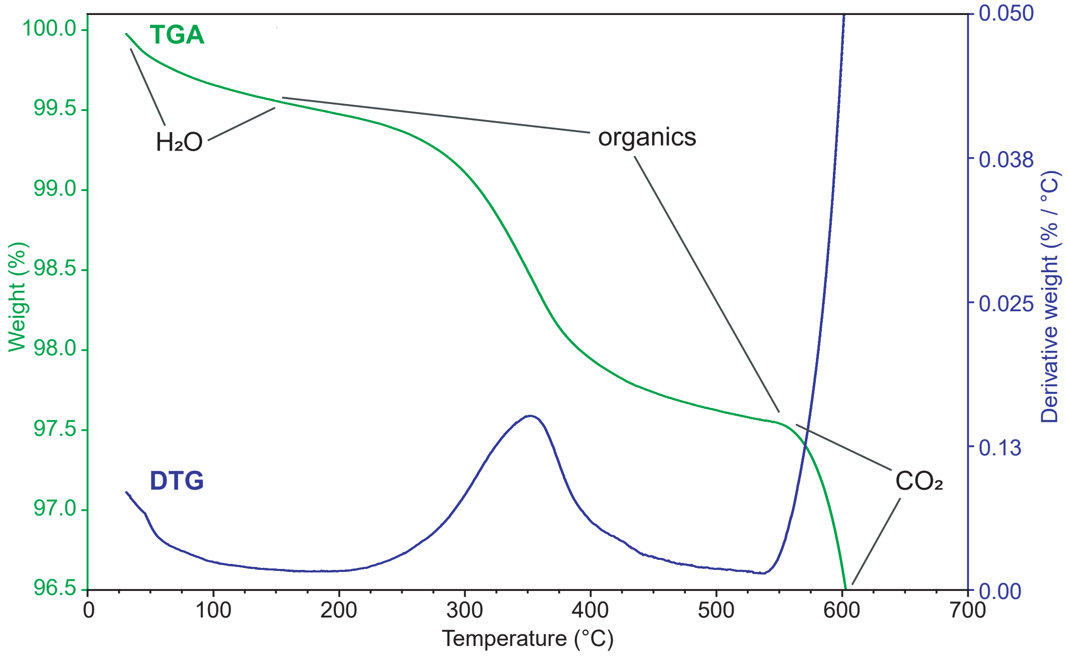

Figure S2 Example of thermogravimetric analysis (TGA, green line) and derivative thermogravimetry (DTG, blue line). The TGA curve represents the weight changes with increasing treatment temperature for the prismatic layer of *Mytilus edulis*. The sample was exposed to a constant heating, from ∼25 °C to 700 °C at a linear rate of 10 °C/min. Three known regions of weight loss with increasing temperature are highlighted (Zaremba et al., 1998)): i) the evaporation of physically adsorbed water at 30 - 150 °C, ii) the degradation of organics at 150 - 550 °C, and iii) the rapid decomposition of calcium carbonate ($CaCO_3$) into calcium oxide (CaO) and carbon dioxide (CO_2) starting at ∼550 °C. The DTG line represents the derivative of the thermal curve and shows the rate of weight loss during heating. The peak indicates the temperature at which the organic mass loss was fastest.

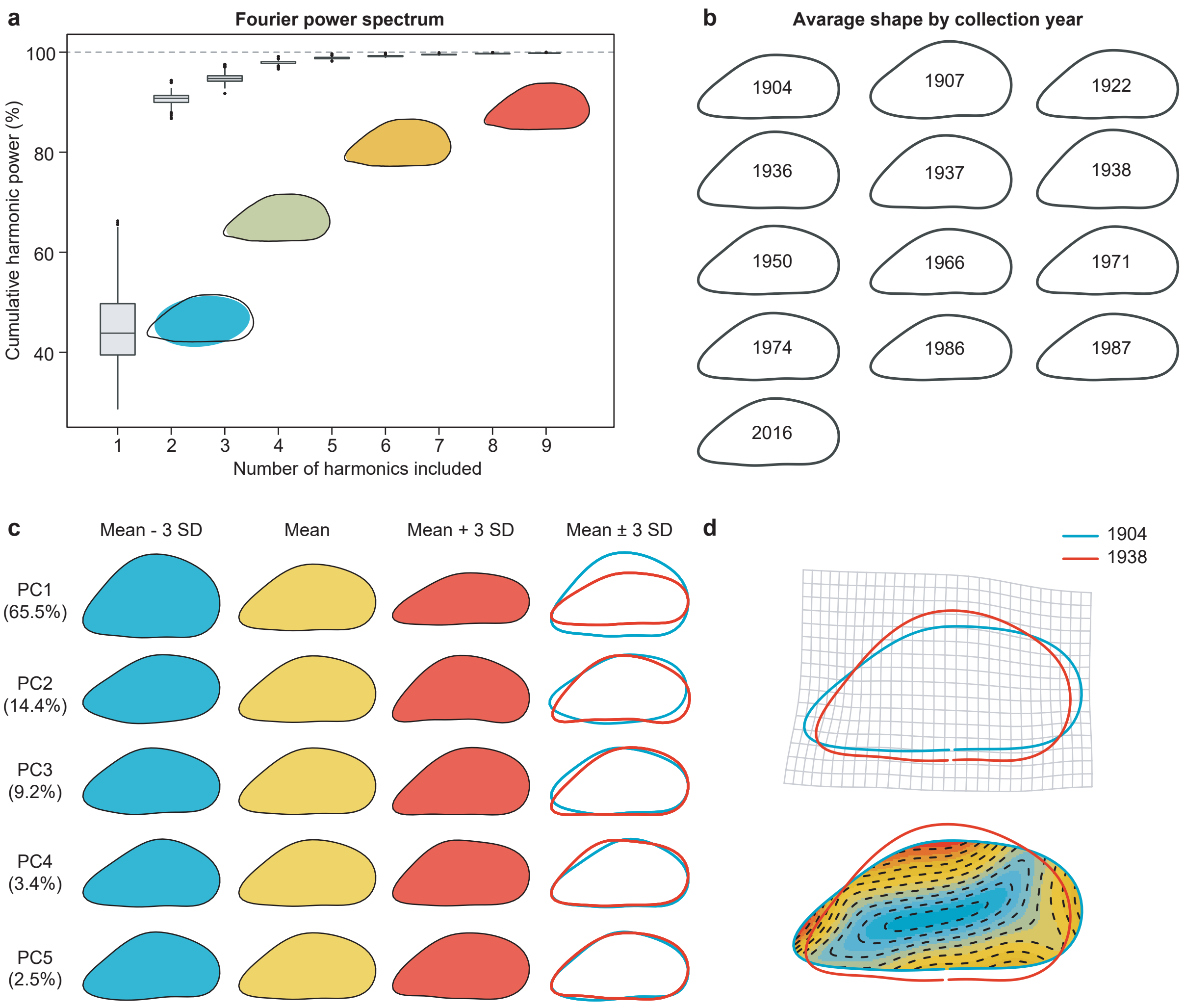

Figure S3 Calibration methods for EFA of shell outlines, and mean shape inspection. (a) Cumulative spectrum of harmonic Fourier power. The power is proportional to the harmonic amplitude and can be considered as a measure of shape information. We evaluated the appropriate number of harmonics to retain (7), so that their cumulative power gathered 99% of the total cumulative power (Crampton, 1995). *Mytilus edulis* average shell outline reconstruction for different numbers of harmonics (1, 4, 6 and 9). Six harmonics gave a satisfactory reconstruction of shell outlines and for nine harmonics the approximation was almost perfect. **(b)** Average shell shapes (left valve) for each collection year. **(c)** Contribution of the first five shape variables (PCs) to shape variation. The average shell shapes for the lateral view were represented for increasing values along each PC (Mean − 3SD, Mean, Mean + 3SD), and extreme shapes were compared (Mean ± 3 SD). **(d)** Differences between mean shapes at the extremes of the morphospace were represented through deformation grids (left), depicting the bindings required to pass from an extreme to another (1904 → 1938), and iso-deformation lines (right), representing the outline regions subjected to different degrees of change (blue: low deformation; red: strong deformation).

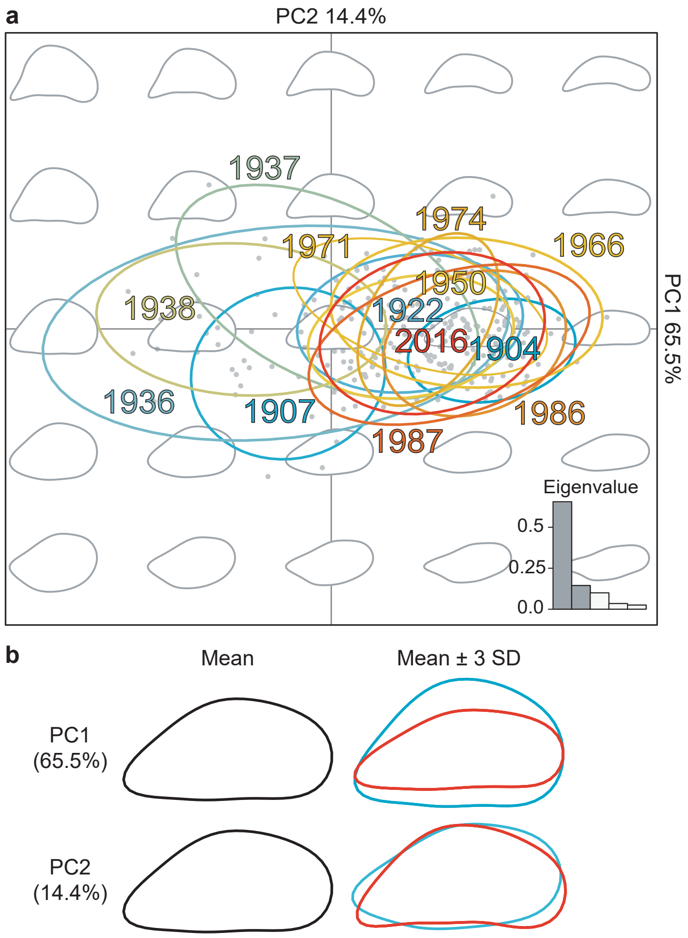

Figure S4 Among-year variation in blue mussel shell shape. **(a)** Scatterplot of the first two principal components (PCs) from a PCA performed on elliptic Fourier coefficients from an EFA, showing significant among-year differences across the morphospace (background) (MANOVA: $n = 268$, Wilk's $\lambda = 0.094$, approximate-$F_{12,242} = 6.64$, $p < 0.0001$). Mussels from 1907 and 1936-1938 had rounder shells than other collection years with more elliptic profiles (Figure S3). Ninety-five per cent confidence intervals (continuous lines) and the proportion of variance explained by the first five PCs (histogram) are reported. **(b)** PCs contribution to the mussel shape variation (Mean + 3SD, red; Mean − 3SD, blue). PC1 (65.5%) captured variations in shell height and ligament angle, with a transition from round to elongated shell for increasing values. This was significantly correlated with shell length ($t_{251} = 3.01$, $p = 0.003$), indicating shape changes during growth (age). PC2 (14.4%) indicated more concave ventral shell profiles for increasing values capturing variations observed under changeable food regimes (Chl-a concentration) (Telesca et al., 2018).

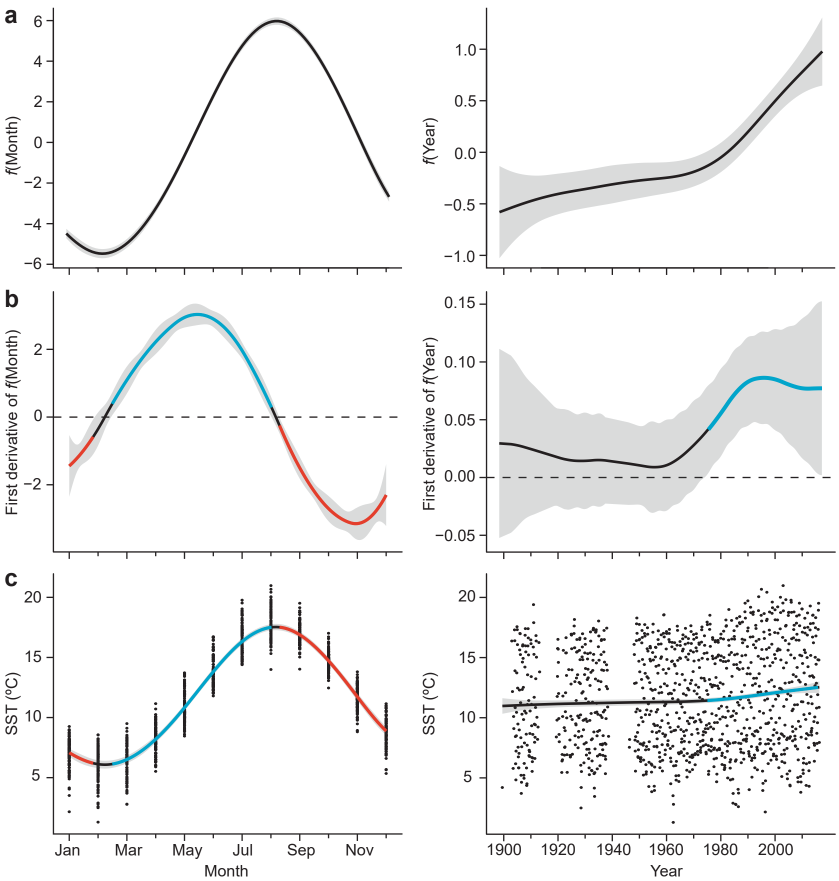

Figure S5 Periods of change in the time series, a worked example. **(a)** Smooth terms for the optimal SST model. The seasonal term (cyclic cubic regression spline) (left) and the trend term (cubic regression spline) (right), indicating long-term changes in the mean level of the time series between 1900 and 2016. Note the very different scales of the two splines, which illustrate the relative degrees of variation in the seasonal (∼12 °C, within-year variation) and trend term (∼2 °C, between-year variation). **(b)** First derivatives for the fitted seasonal and trend splines estimated with the method of finite differences. This method approximates the first derivative of a fitted spline by i) choosing a set of points p on the function and another set p' positioned at a very small distance from the first set, ii) evaluating the fitted trend spline at the location p and p', and iii) computing the rate of change (slope) in the function between the pair of points. The shaded areas represent simultaneous 95% confidence intervals, reflecting the uncertainty of the fitted functions. These were calculated through a posterior simulation-based approach following Ruppert et al. (2003) (see Supplementary Methods). Sections of the spline derivatives where zero is not included in the 95% simultaneous confidence interval represent periods of significant change in the time series (blue: increase; red: decrease). **(c)** Intervals of significant change are then superimposed to the fitted trends to show period of significant increase or decrease for the seasonal and long-term SST patterns.

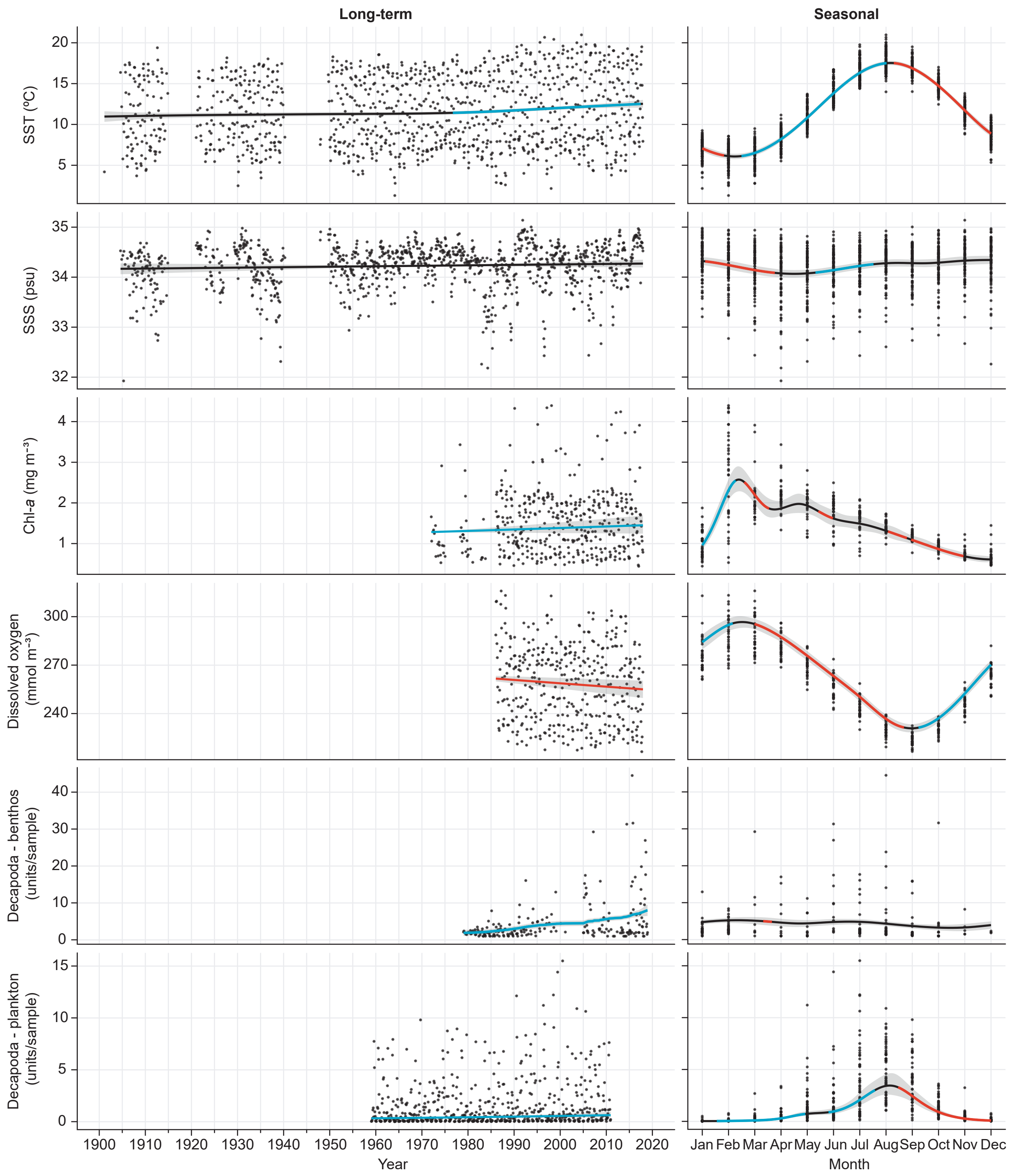

Figure S6 Temporal variation of annual mean and seasonal environmental conditions. Predictions of long-term variation and seasonal patterns of key environmental descriptors and the abundance of decapods along the Belgian coastline over the last century. From top to bottom: SST, SSS, Chl-a concentration, dissolved oxygen, abundance of benthic decapods and their planktonic stages. Periods of significant increase (blue) and decrease (red) in the predicted trends (solid lines), and the 95% simultaneous confidence intervals (shaded area) are reported.

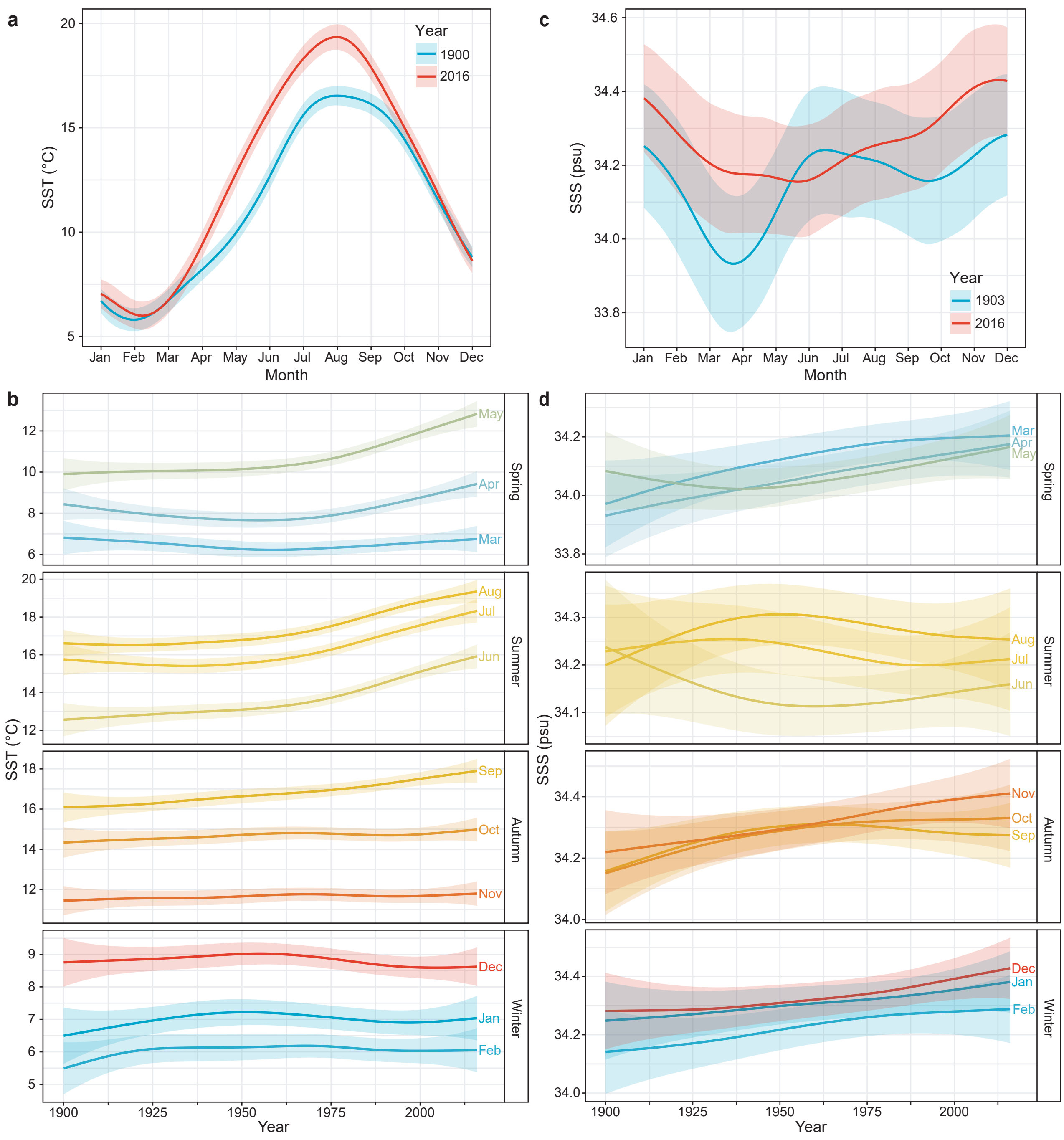

Figure S7 Historical variation in the seasonal pattern (within-year variation) for SST and SSS. (a) Predicted seasonal (monthly) variation for 1900 and 2016 (13.6 °C within-year variation), and **(b)** trends (long-term variations) for each month grouped by quarter/season for SST over the 1900 - 2016 period. SST increased in spring and summer (April - September) with maxima of +2.9 - 3.6 °C in May and August, and no variation in winter and autumn. **(c)** Predicted seasonal variation for 1904 and 2016 (0.6 psu within-year variation), and **(d)** trends for each month grouped by quarter/season for SSS over the 1904 - 2016 period. SSS increased in autumn (October-November) and early spring (February-April), with maximum mean change of 0.25 psu in March and April. Simultaneous 95% confidence intervals (shaded areas) are reported for each prediction (solid lines).

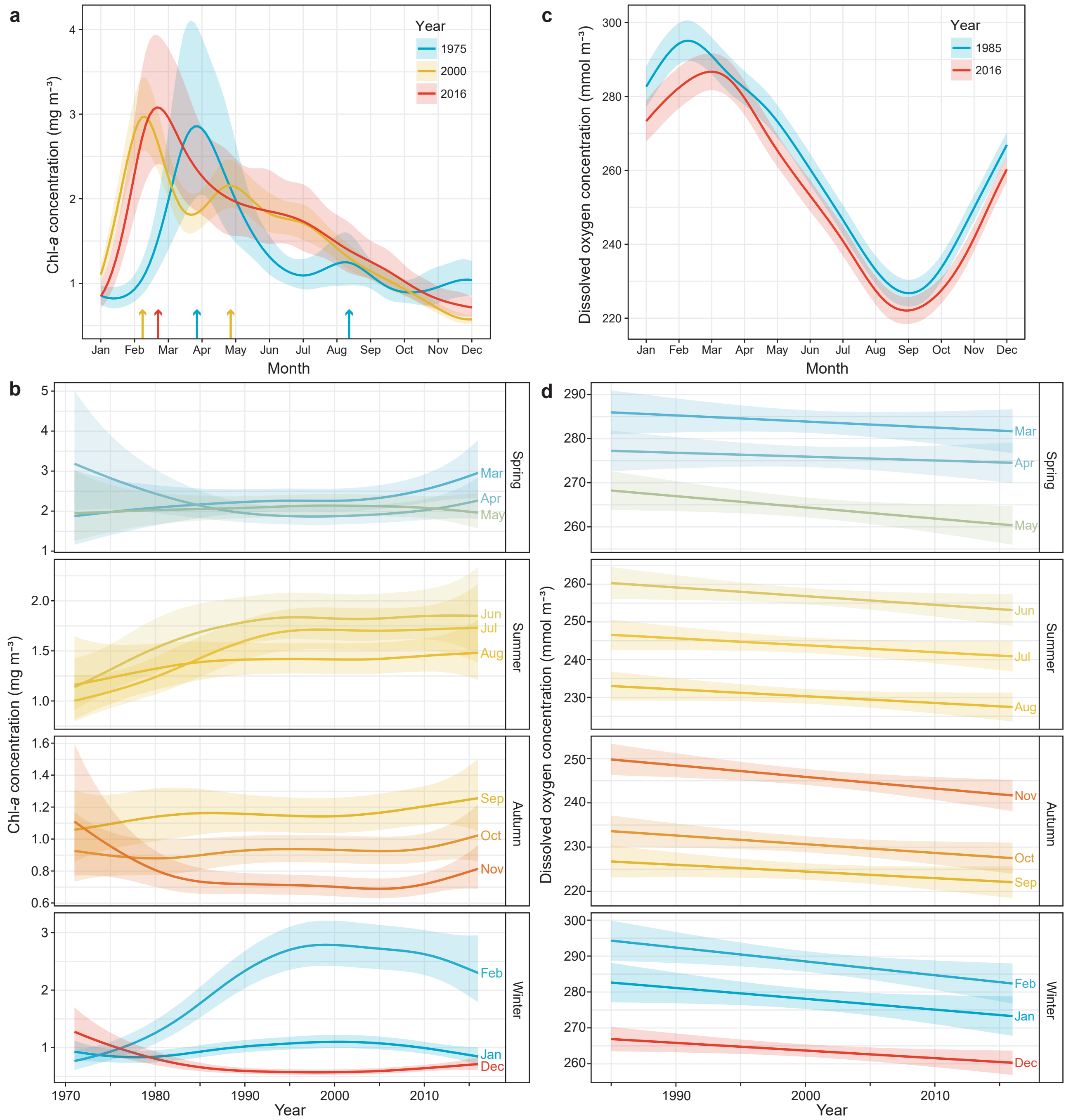

Figure S8 Historical variation in the seasonal pattern (within-year variation) for Chl-*a* and dissolved oxygen concentration. **(a)** Predicted seasonal (monthly) variation for 1975, 2000 and 2016 (2.5 mg m^3 within-year variation), and **(b)** trends (long-term variations) for each month grouped by quarter/season for surface Chl-*a* concentration over the 1975 - 2016 period. **(c)** Predicted seasonal variation for 1985 and 2016 (65.82 mmol m^3 within-year variation), and **(d)** trends for each month grouped by quarter/season for surface dissolved oxygen over the 1985 - 2016 period. Dissolved oxygen decreased all-over the year, except in March-April, with a maximum of -11.9 mmol m^3 in winter (February). Simultaneous 95% confidence intervals (shaded areas) are reported for each prediction (solid lines).

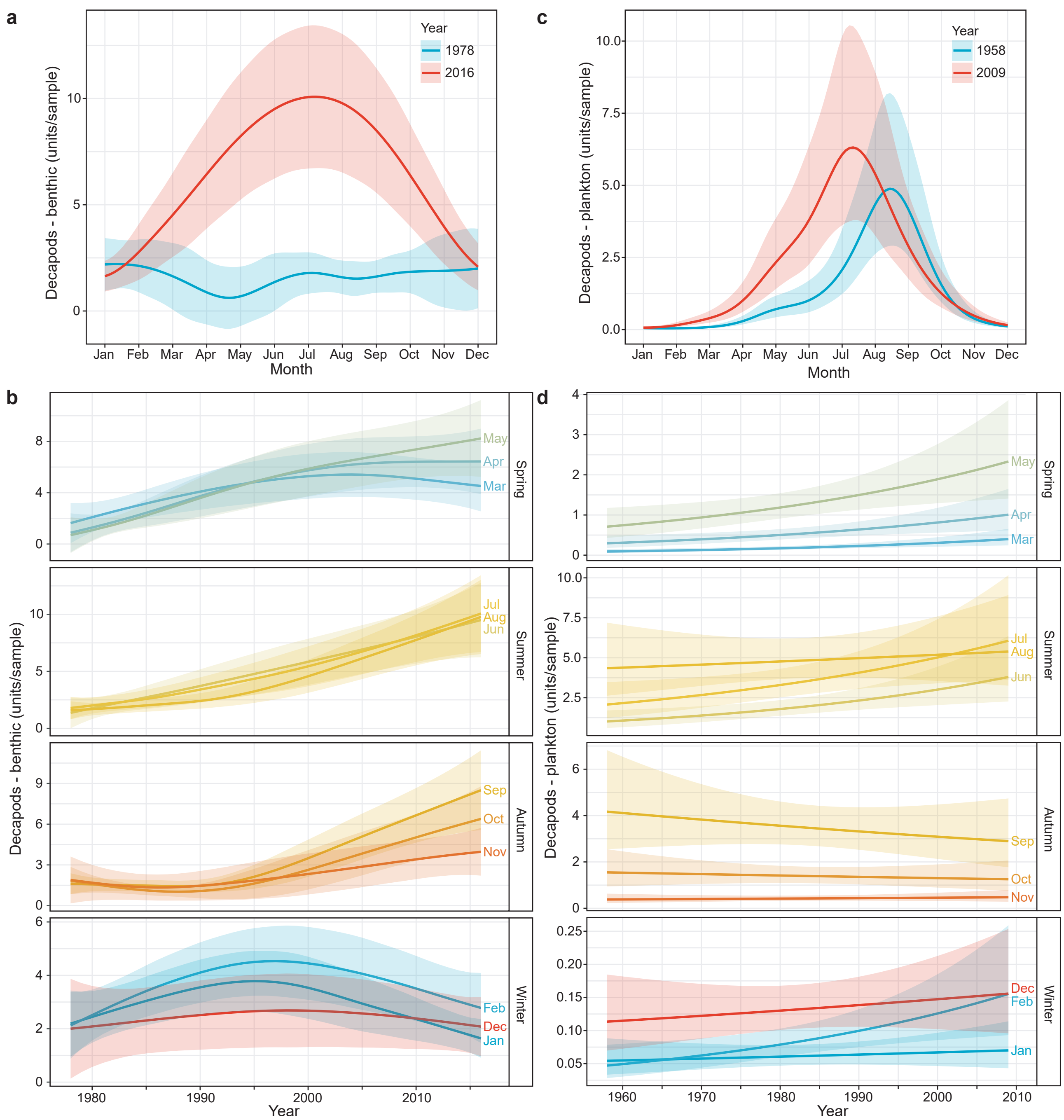

Figure S9 Historical variation in the seasonal pattern (within-year variation) for benthic decapods and their planktonic larvae. **(a)** Predicted seasonal variation for 1978 and 2016 (10 units/sample within-year variation), and **(b)** trends for each month grouped by quarter/season for the abundance of macrobenthic decapods over the 1978 - 2016 period. **(c)** Predicted seasonal (monthly) variation for 1978 and 2009 (6 units/sample within-year variation), and **(d)** trends (long-term variations) for each month grouped by quarter/season for the abundance of decapod planktonic larvae over the 1978 - 2009 period. Simultaneous 95% confidence intervals (shaded areas) are reported for each prediction (solid lines).

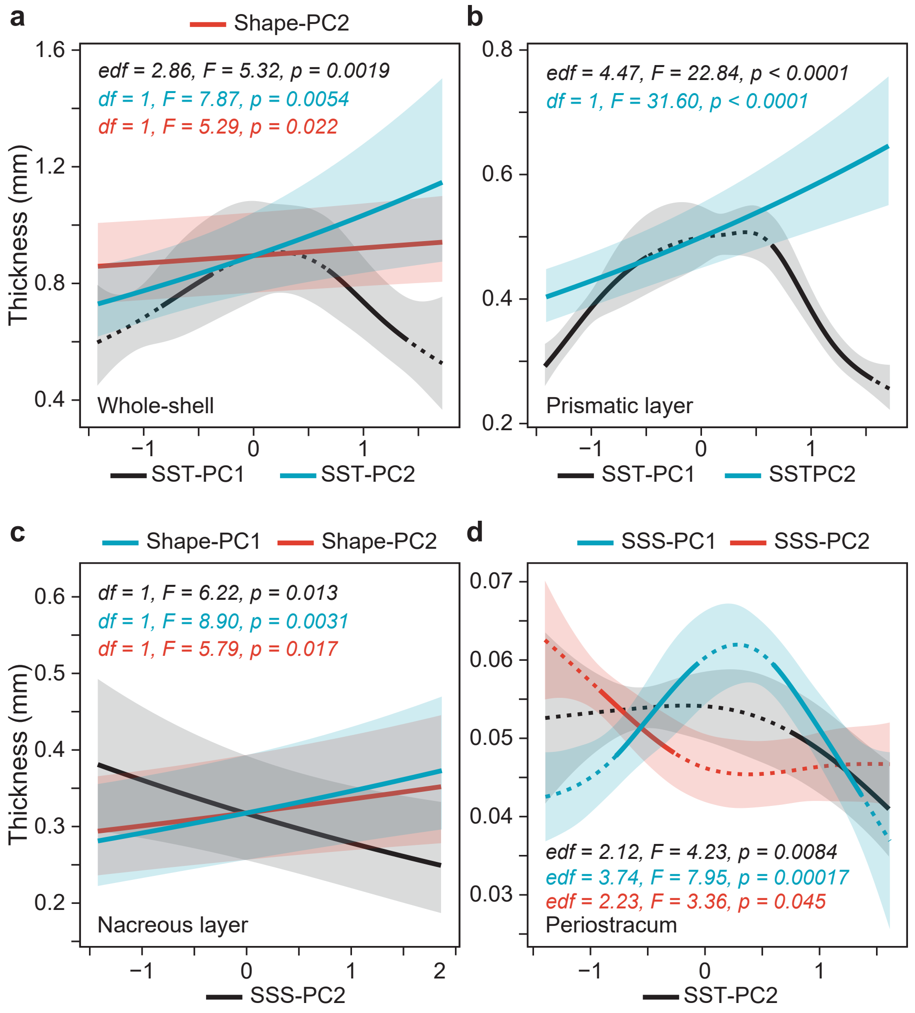

Figure S10 Environmental effects on blue mussel shell layers. **(a)** Whole-shell thickness increased with increasing food supply (Shape-PC2) and under more variable SST regimes (SST-PC2), while decreased under low and high mean SSTs (SST-PC1). **(b)** The thickness of prismatic layer increased under more variable SST regimes (SST-PC2), while decreased under low and high mean SSTs (SST-PC1). **(c)** The nacreous layer thickness increased with increasing food supply (Shape-PC2), and decreased under more variable SSS regimes (SSS-PC2). **(d)** Periostracum thickness increased non-linearly under average salinities (SSS-PC1) and less variable SSS regimes (SSS-PC2), while decreased under more variables SST regimes (SST-PC2). Periods of significant and non-significant change (solid and dashed lines, respectively), and 95% simultaneous confidence intervals (shaded area) are reported. Details in Table S7.

Table S1 Collection details for archival and current *Mytilus edulis* shells used for the study. For each sampling site (site code as in Figure S1), the year of collection, geographic location, site coordinates (latitude and longitude), sample size (N), average sample shell length, and collection type (wet: shell + tissue; dry: shell only; live: hand-collected mussels) are reported. All the blue mussel specimens were collected from the intertidal zone on stone breakwaters.

**Site**	**Year**	**Location**	**Latitude (N)**	**Longitude (E)**	N	**Length ± SD**	**Collection type**
1	1904	Westende	51.168751	2.762271	25	55.70 ± 4.01	Wet
2	1907	Oostende - Turkije	51.240798	2.929859	13	47.64 ± 6.71	Wet/Dry
3	1922	Westende - Middelkerke	51.183889	2.798636	25	53.56 ± 5.72	Dry
4	1936	Westende - Middelkerke	51.194368	2.823377	15	54.08 ± 5.58	Wet/Dry
5	1937	Westende - Middelkerke	51.190883	2.815409	12	55.38 ± 4.78	Wet
6	1938	Westende - Middelkerke	51.194658	2.822695	15	53.97 ± 3.44	Wet/Dry
7	1950	Raversijde	51.206001	2.849758	25	53.97 ± 3.44	Wet
8	1966	Westende	51.170646	2.766574	13	54.17 ± 5.61	Wet
9	1971	Mariakerke	51.224318	2.889219	22	51.21 ± 3.89	Wet
10	1974	Raversijde	51.206325	2.849672	26	54.31 ± 3.15	Wet
11	1986	Westende - Middelkerke	51.182291	2.794256	26	55.35 ± 2.84	Wet
12	1987	Westende - Middelkerke	51.181848	2.794758	21	51.97 ± 4.55	Wet
13	2016	Mariakerke	51.228793	2.898682	30	47.38 ± 2.03	Live

Table S2 List of key packages used with the R software for data exploration, statistical analysis and graphing.

Package	Complete name	Version	Year	Author	Use
betareg	Beta Regression	3.1-1	2018	Cribari-Neto & Zeileis (2010)	Beta regression (GLM with beta distribution)
car	Companion to Applied Regression	3.0-2	2018	Fox & Weisberg (2011)	Type III ANOVA table
gamm4	Generalized Additive Mixed Models using 'mgcv' and 'lme4'	0.2-5	2017	Wood & Scheipl (2017)	Generalized additive (mixed) models (GA(M)Ms) using an lme4 method
ggplot2	Create Elegant Data Visualisations Using the Grammar of Graphics	3.1.0	2018	Wickham (2016)	Graphing
glmmTMB	Generalized Linear Mixed Models using Template Model Builder	0.2.3	2019	Brooks et al. (2017)	Mixed-effect models (GLMMs) using the template model builder
lme4	Linear Mixed-Effects Models using 'Eigen' and S4	1.1-21	2019	Bates et al. (2015)	Mixed-effect models (GLMMs)
lmerTest	Tests in Linear Mixed Effects Models	3.1-0	2019	Kuznetsova et al. (2017)	Wald approximated confidence intervals
mgcv	Mixed GAM Computation Vehicle with Automatic Smoothness Estimation	1.8-27	2019	Wood (2017)	Generalized additive (mixed) models (GA(M)Ms)
Momocs	Outline analysis using R	1.29	2018	Bonhomme et al. (2014)	Elliptic Fourier analysis of shell outlines
MuMIn	Multi-Model Inference	1.42.1	2018	Barton (2017)	Pseudo-R-squared for Generalized Mixed-Effect models
nlme	Linear and Nonlinear Mixed Effects Models	3.1-137	2018	Pinheiro et al. (2017)	Mixed-effect models (GLMMs), variance structure (GLS), spatial correlation
tsgam	Utilities for Working with GAMs Fitted to Time Series	0.0-4	2018	Simpson (2018)	Derivative, simultaneous confidence interval and simulations estimation for time series analyses with GAMMs (now integrated in the package "schoenberg")

Table S3 Environmental descriptors and their loadings on the first two PCs from PCAs performed on SST- and SSS-related variables.

	SST		SSS	
**Descriptor**	**PC1**	**PC2**	**PC1**	**PC2**
Mean	**-0.44**	0.16	**0.42**	-0.08
Median	**-0.40**	0.19	**0.40**	0.13
SD	0.23	**0.46**	-0.24	**0.57**
Max	0.05	**0.49**	-0.10	**-0.58**
Min	**-0.40**	-0.13	**0.29**	0.04
90%	-0.11	**0.51**	0.26	**0.41**
75%	-0.29	**0.38**	**0.38**	0.27
25%	**-0.43**	-0.11	**0.40**	-0.19
10%	**-0.39**	-0.23	**0.38**	-0.22

Table S4 GAMMs summary statistics for *Mytilus edulis* shell thickness and shape variation with collection year and shell size. Variation of whole-shell, prismatic layer, nacreous layer and periostracum thickness (n = 256), and shell shape (shape-PC1 - PC2, n = 265; Figure S4) with year of collection (Year) and shell size (Length).

Parameter	edf	Estimate (SE)	z	F	p-value	Random effects	SD
**Whole-shell**							
(Intercept)	1	6.402 (0.091)	70.26	_	<**0.0001**	Site	0.163
Year	1	0.004 (0.001)	2.75	_	**0.0059**	Residual	0.190
Length	1	0.002 (0.003)	0.58	_	0.56		
**Prismatic layer**							
(Intercept)	1	5.994 (0.020)	_	93952.67	<**0.0001**	Site	0.095
f(Year)	4.04	_	_	23.70	<**0.0001**	Residual	0.009
Length	1	0.009 (0.003)	_	10.20	**0.001**		
**Nacreous layer**							
(Intercept)	1	5.568 (0.121)	45.98	_	<**0.0001**	Site	0.210
Year	1	0.005 (0.002)	2.58	_	**0.010**	Residual	0.335
Length	1	-0.003 (0.005)	-0.67	_	0.50		
**Periostracum**							
(Intercept)	1	3.895 (0.025)	_	24567.43	<**0.0001**	Site	0.066
f(Year)	3.51	_	_	17.58	<**0.0001**	Residual	0.261
Length	1	0.001 (0.004)	_	0.04	0.84		
**Shape-PC1**							
(Intercept)	1	-0.001 (0.001)	_	2.96	0.11	Site	0.001
Year	1	1.6e-05 (9.3e-06)	_	2.86	0.12	Residual	0.001
Length	1	4.1e-05 (1.4e-05)	_	9.06	**0.0029**		
**Shape-PC2**							
(Intercept)	1	-1.4e-04 (1.6e-04)	_	0.79	0.39	Site	0.001
Year	1	2.6e-06 (2.5e-06)	_	1.10	0.32	Residual	0.001
Length	1	6.9e-06 (8.9e-06)	_	0.59	0.44		

Table S5 GAMMs summary statistics (time-series analyses) of environmental descriptors and abundance of predators. Summary statistics and the significance of the trend term [f(Year), long-term change], seasonal pattern, [f(Month), within-year variation], and the interaction between seasonal and trend terms, [f(Year × Month), smooth tensor product interaction] are reported.

**Parameter**	**edf**	**Estimate (SE)**	***F***	***p*-value**
**SST**				
(Intercept)	1	11.55 (0.06)	40040.01	<**0.0001**
f(Year)	2.90	–	17.39	<**0.0001**
f(Month)	8.97	–	894.05	<**0.0001**
f(Year × Month)	39.81	–	0.97	<**0.0001**
**Salinity**				
(Intercept)	1	32.420 (1.654)	384.32	<**0.0001**
Year	1	0.001 (0.001)	1.19	0.28
f(Month)	4.98	–	5.35	<**0.0001**
f(Year × Month)	7.95	–	0.05	0.12
**Chl-*a***				
(Intercept)	1	-7.308 (2.286)	10.22	**0.0015**
Year	1	0.004 (0.001)	11.1	**0.00094**
f(Month)	8.71	–	143.24	<**0.0001**
f(Year × Month)	28.62	–	0.89	<**0.0001**
**Dissolved oxygen**				
(Intercept)	1	676.238 116.358	33.78	<**0.0001**
Year	1	-0.209 (0.058)	12.90	**0.00037**
f(Month)	8.14	–	225.60	<**0.0001**
f(Year × Month)	0.01	–	0.01	0.72
**Decapods (benthos)**				
(Intercept)	1	4.466 (0.343)	169.00	<**0.0001**
f(Year)	2.36	–	19.87	<**0.0001**
f(Month)	3.64	–	1.33	**0.00016**
f(Year × Month)	4.18	–	0.12	**0.0072**
**Decapods (plankton)**				
(Intercept)	1	-26.565 (7.378)	12.97	**0.00034**
Year	1	0.013 (0.004)	12.54	**0.00043**
f(Month)	7.46	–	92.46	<**0.0001**
f(Year × Month)	9.48	–	0.12	**0.00070**

Table S6 GAM summary statistics (parametric and smooth terms) of breeding pair numbers variation with seagull species and collection year, and their interaction.

Parameter	edf	Estimate (SE)	F	p-value
**Parametric coefficients**				
(Intercept)$^{+}$	1	8.651 (0.081)	11344.38	<**0.0001**
Species(Herring)	1	-4.524 (0.100)	2058.26	<**0.0001**
Species(Lesser Black-Backed)	1	-5.316 (1.463)	13.21	**0.00042**
Species(Common)	1	-7.137 (0.230)	963.23	<**0.0001**
**Approximate significance of smooth terms**				
f(Year) × Species(Black-Headed)	6.02	_	11.74	<**0.0001**
f(Year) × Species(Herring)	7.65	_	624.91	<**0.0001**
f(Year) × Species(Lesser Black-Backed)	3.96	_	399.31	<**0.0001**
f(Year) × Species(Common)	5.12	_	75.06	<**0.0001**

$^{+}$Species(Black-Headed) is used as the intercept.

Table S7 GAMMs summary statistics for *M. edulis* shell thickness variation with environmental regimes and shell shape.

Parameter	edf	Estimate (SE)	F	p-value	Random effects	SD
**Whole-shell**						
(Intercept)	1	6.591 (0.040)	27278.820	<**0.0001**	Site	0.135
Shape-PC2	1	0.030 (0.013)	5.290	**0.022**	Residual	0.189
f(SST-PC1)	2.86	–	5.318	**0.0019**		
SST-PC2	1	0.145 (0.052)	7.868	**0.0054**		
**Prismatic layer**						
(Intercept)	1	5.967 (0.020)	92610.050	<**0.0001**	Site	0.055
f(SST-PC1)	4.47	–	22.840	<**0.0001**	Residual	0.188
SST-PC2	1	0.151 (0.027)	31.596	<**0.0001**		
Length	1	0.035 (0.013)	7.049	**0.0085**		
**Nacreous layer**						
(Intercept)	1	5.777 (0.046)	15764.810	<**0.0001**	Site	0.145
Shape-PC1	1	0.086 (0.029)	8.898	**0.0031**	Residual	0.337
Shape-PC2	1	0.055 (0.023)	5.794	**0.017**		
f(SST-PC2)	3.77	–	4.700	**0.0021**		
**Periostracum**						
(Intercept)	1	50.631 (1.048)	2333.856	<**0.0001**	Site	2.311
f(SST-PC2)	2.12	–	4.231	**0.0084**	Residual	13.01
f(SSS-PC1)	3.74	–	7.948	**0.00017**		
f(SSS-PC2)	2.23	–	3.359	**0.045**		

Table S8 Summary statistics for thickness variation of each shell layer in *Mytilus edulis* with different predation regimes.

Predator	Contrast	Difference (SE)	Lower	Upper	t	p-value
**Prismatic layer**						
Decapods	High vs Low	0.121 (0.031)	0.060	0.181	3.939	**0.0001**
Seagulls	High vs Low	0.225 (0.028)	0.169	0.281	7.934	<**0.0001**
Dog whelks	Presence vs Absence	0.086 (0.021)	0.044	0.128	4.003	**0.0001**
**Nacreous layer**						
Decapods	High vs Low	0.104 (0.038)	0.030	0.177	2.760	**0.0061**
Seagulls	High vs Low	0.112 (0.050)	0.012	0.211	2.220	**0.028**
Dog whelks	Presence vs Absence	-0.003 (0.027)	-0.056	0.051	-0.109	0.91
**Periostracum**						
Decapods	High vs Low	-0.029 (0.004)	-0.037	-0.020	-6.678	<**0.0001**
Seagulls	High vs Low	-0.023 (0.005)	-0.032	-0.014	-4.921	<**0.0001**
Dog whelks	Presence vs Absence	0.018 (0.003)	0.012	0.024	5.817	<**0.0001**

Supplementary Methods

Physical environment: the Belgian coastline

The Belgian coastline is a 65-km long, southwest to northeast directed, almost linear sandy shoreline between 51°05'N-02°32'E and 51°05'N-03°22'E (Figure S1). The sublittoral habitat is a shallow sandy bottom, making this coastal profile unsuitable for mussel beds or other rocky shore organisms. However, intensive human activities during the past 200 years have provided suitable substrata for hard bottom benthic assemblages, such as dikes, harbour-related infrastructures, and breakwaters (Warmose et al., 1988).

Among these, a series of regularly placed stone breakwaters was built to stabilise Belgian beaches against high levels of coastal erosion (Figure S1). Breakwaters are perpendicular to the coastline and constructed of basalt rocks or concrete. Their length is $\sim$400 m being in large part situated in the intertidal zone. The distance between two consecutive breakwaters is 200-500 m. According to Becuwe (1971), the first breakwaters were built between 1815 and 1830, and many of those still in place were built around 1880.

Protocol for Thermal Gravimetric Analyses

Protocol for Thermal Gravimetric Analyses (TGA) are reported following the guidelines made by the Committee on Standardisation of the International Confederation for Thermal Analysis and Calorimetry (ICTAC) and appeared in standards as ASTM E 472 (1991) (P. J. Haines, 2002; Gaisford et al., 2016).

A) Properties of the sample

i) *Source of material and identification*
- Shell of wild Atlantic blue mussel (*Mytilus edulis*).
- Prismatic layer composed of calcium carbonate ($CaCO_3$, calcite), variable amount of organics (1 - 2%) and other components, such as quartzite (SiO_2) and magnesium (Mg).

ii) *Sample history*
- Shells were cleaned, raised with mill-Q water, dried at room temperature for seven days.
- The periostracum was removed by sanding and a tile of prismatic layer isolated (8 × 5 mm) with a Dremel rotary tool (Dremel 300/395RD MultiPro, Racine, Wisconsin, USA).
- Samples were cleaned in an ultrasonic bath (Ultrasonic Cleaner CD-4800, Practical Systems Inc., Odessa, FL, USA) with mill-Q water, air-dried and powdered with an agate mortar.
- Additional, oven drying (30 °C for 24h, convection oven) to remove residual pre-treatment water.

iii) *Physical properties*
- Fine grade powder.

B) Experimental conditions

i) *Apparatus used*
- Thermogravimetric Analyser: TGA Q500, TA instrument (New Castle, DE, USA) Q series.

ii) *Thermal treatment*
- Initial temperature, ∼25 °C (room temperature).
- Final temperature, 700 °C.
- Linear rate of heating, 10 °C min^{-1}.

iii) *Sample atmosphere*
- Dynamic (flowing) atmosphere.
- Flow rate for balance 40 ml $min^{-1}$and for sample 60 ml min^{-1}.
- Gas composition: nitrogen, "white spot".

iv) *Sample holder*
- Platinum crucible, cylindrical: diameter 10 mm and height 1.5 mm.
- Sample was tipped and spread to cover the bottom of the crucible.

v) *Sample mass*
- 10 mg of powder were weighted on a separate micro-balance (Ultramicro 4504 MP8, Sartorius, Göttingen; readability 0.1 µg).

C) Data acquisition and manipulation methods

i) *Software version*
- Universal Analysis 2000, version 4.5A, TA instrument (New Castle, DE, USA).

Geometric morphometrics analysis

Mytilus edulis shell shape was analysed through an elliptic Fourier analysis (EFA) of outlines (Giardina & Kuhl, 1977; Kuhl & Giardina, 1982; Bonhomme et al., 2014). This geometric morphometrics approach (Rohlf & Marcus, 1993; Adams et al., 2004) was performed to examine shell shape variation both within and between groups of individuals. As other modern morphometrics approaches, it considers outlines as a whole, taking into account all the geometrical relationships of the input data.

EFA is a powerful method to extract geometric information and has been implemented on the concept of Fourier series: to decompose a periodic function into a sum of more simple trigonometric functions, such as sine and cosine (Claude, 2008; Bonhomme et al., 2014). These simple functions have frequencies that are integer multiples, therefore they are harmonics of one another.

This approach fits Fourier series separately on the x and y coordinates of an outline, projected on the Cartesian plane, as a function of the curvilinear abscissa (Kuhl & Giardina, 1982; Claude, 2008; Bonhomme et al., 2014). EFA is then used to extract the geometrical information from outlines, described as periodic functions (Kuhl & Giardina, 1982; Rohlf & Archie, 1984), through their decomposition into the harmonic sum of trigonometric functions, called harmonics. Low-frequency harmonics approximate coarse-scale trends in the original outlines, while high-frequency harmonics fit their fine-scale variations (Bonhomme et al., 2014). Outlines can be normalized to remove homothetic, translational or rotational differences between shapes and smoothed to remove outline noise (A. J. Haines & Crampton, 2000). Harmonic coefficients are then extracted and used as shape variables. The geometrical information contained in the outlines is thus quantified and can be analysed with classical multivariate tools (i.e. multivariate analysis of variance, principal component analysis, and linear discriminant analysis).

EFA of outlines allows shape reconstruction from the numerical signature and this improved method has great advantages compared to more traditional approaches (A. J. Haines & Crampton, 2000; Adams et al., 2004; Bonhomme et al., 2014): complex shapes can be fitted, outlines smoothed, starting points and coefficients can be normalised to remove homothetic, translational and rotational differences between outlines (Rohlf & Archie, 1984; Crampton, 1995; A. J. Haines & Crampton, 2000; Adams et al., 2004; Bonhomme et al., 2014). EFA were carried out using the Momocs (v1.2.9, “Morphometrics using R”) (Bonhomme et al., 2014) package with the R v3.5.2 software (R Core Team, 2016).

EFA of outlines: acquisition, processing and analysis (Telesca et al., 2018)

- Digital images of lateral shell views (left valves) were acquired with a high-resolution digital camera (Nikon D3300 camera, fitted with Sigma 105mm f/28 EX DG Macro lens);
- Photographs were processes with an image analysis software (©Adobe Photoshop), centred and consistently aligned;
- Photographs were converted into black masks on a white background (greyscale, 8-bit) and only the shapes of intact shells were retained;
- Outlines were isolated, converted into a list of $(x; y)$ pixel coordinates and used as input data.

Outlines were processed prior to calculation of elliptic Fourier transforms:

- An outline alignment through geometric operations was directly performed on the list of coordinates. This *a priori* normalisation was required to avoid potential bias introduced by the numerical adjustment of shapes prone to bad alignment (usually circular or with bilateral symmetry). Indeed, for mussel outlines, “consuming” their first harmonic to normalise higher rank harmonics (Claude, 2008), determined a poor numerical alignment, resulting in not homologue elliptic Fourier descriptors (Rohlf & Archie, 1984; A. J. Haines & Crampton, 2000);

- Outlines were first smoothed to remove any noise introduced during the digitization process, centred, and outline coordinates were rescaled by their centroid size;
- Equal number of points were sampled along each outline (1,000 pseudo-landmarks);
- Point configurations were aligned through a Procrustes superimposition (Bookstein, 1991; Claude, 2008) and starting points normalised;
- An EFA was then computed on the resulting coordinates from shapes invariant to outline size, rotation, and position;
- After preliminary calibration, through inspection of i) the outline reconstruction efficiency, ii) the deviation from the optimal fit, and iii) the spectrum of harmonic Fourier power, seven harmonics were chosen to encompass 99% of the total harmonic power (Crampton, 1995; Bonhomme et al., 2014);
- Four coefficients per harmonic (28 descriptors) were extracted for each outline and used as variables quantifying the geometrical information (Rohlf & Archie, 1984; Claude, 2008).

The shape information contained in the outlines was then quantified and analysed with classical multivariate tools:

- Principal component analysis (PCA), with a singular value decomposition method, was performed on the matrix of coefficients, without rescaling, to define axes capturing the most of the shape variation among individuals (Claude, 2013; Telesca et al., 2018);
- Multivariate analysis of variance (MANOVA) was performed on the new shape variables to test for a significant effect of collection year and shell size on shape variances (Claude, 2008, 2013);
- Shape differences were visualised with representation of mean shapes, deformation grids (Thompson, 1917) and iso-deformation lines, though mathematical formalisation of thin plate splines (TPS) analysis (Bookstein, 1991).

Model predictions

To identify periods of statistically significant change in the time series analysed with GAMMs, we first used the method of finite differences to compute the first derivative of the fitted splines (Figure S5). Without an equation for the spline, a derivative cannot be calculated analytically. Hence, this method samples a number of infinitesimally distant points on the fitted spline, and estimates the slope between pairs of points throughout the trend. The first derivative of the spline is then approximated to the change in the slopes between these points. Following Ruppert et al. (2003), we then used a posterior simulation-based approach to generate simultaneous 95% confidence intervals drawing from the Bayesian covariance matrix of the model derivative (10,000 draws). This approach calculates the appropriate critical value (scaling factor of the standard error) to estimate a 95% confidence interval reflecting the uncertainty of the fitted function. Estimated points of the spline's derivative at which the simultaneous 95% confidence interval does not include zero represent periods of significant change in the time series.

Supplementary Data

Environmental Descriptors

Environmental datasets (1900-2016)

Historical data for SST (1900 - 1984), SSS (1904 - 1984) were obtained from the International Council for the Exploration of the Sea (ICES) Data Centre (http://www.ices.dk/marine-data/) (ICES, 2004) and the Integrated Marine Environmental Readings and Samples (IMERS) (IMERS, 2018) (http://www.vliz.be/vmdcdata/imers/). Chl-a concentration (1971 - 1984) datasets were obtained from the Management Unit of the Mathematical Model of the North Sea (MUMM, at the RBINS, http://www.bmdc.be/), ICES (ICES, 2004) and IMERS (IMERS, 2018).

Environmental datasets (1985-2016)

List of environmental datasets used for SST, SSS, Chl-a concentration and dissolved oxygen. Time-series of daily measurements for each descriptor during the 1985 - 2016 period were generated using the Copernicus Marine Environment Monitoring Service (CMEMS) (EU Copernicus Marine Service, 2018) (http://marine.copernicus.eu/).

DATASET #1

Product identifier **NORTHWESTSHELF_ REANALYSIS_ PHYS_ 004_ 009**

Link http://marine.copernicus.eu/services-portfolio/access-to-products/?option=com _csw&view=details&product_id=NORTHWESTSHELF_REANALYSIS_PHY_004_009 (last accessed on 10-06-2018)

Short description The reanalysis covers the period January 1985 until July 2014 and is based upon the Forecasting Ocean Assimilation Model 7km Atlantic Margin Model (FOAM AMM7).This is a hydrodynamic model of the North West European shelf forced at the surface by ERA-interim winds, atmospheric temperature, and precipitation fluxes. Horizontal boundary conditions were provided by the NOC global reanalysis prior to 1989 and by the GloSea reanalysis thereafter. Boundary conditions in the Baltic sea came from the IOM-GETM model. E-Hype data were used for river inputs. Hydrodynamic calculations were performed by the Nucleus for European Modelling of the Ocean (NEMO) system, while the 3DVar NEMOVAR system was used for the assimilation of sea surface temperature data. Physical outputs are provided both as monthly means and as daily 25 hour, de-tided, averages.

Spatial resolution 0.11 degree × 0.07 degree

Vertical coverage from -5500.0 m to 0.0 m

Temporal resolution Daily mean, monthly mean

Update frequency Daily

Production unit NWS-METOFFICE-EXETER-UK

DATASET #2

Product identifier **NORTHWESTSHELF_ ANALYSIS_ FORECAST_ PHYS_ 004_ 001_ b**

Link `http://marine.copernicus.eu/services-portfolio/access-to-products/?option=com_csw&view=details&product_id=NORTHWESTSHELF_ANALYSIS_FORECAST_PHYS_004_001_b` (last accessed on 10-06-2018)

Short description The Forecasting Ocean Assimilation Model 7km Atlantic Margin model (FOAM AMM7) is a coupled hydrodynamic-ecosystem model, nested in a series of one-way nests to the Met Office global ocean model. The hydrodynamics are supplied by the Nucleus for European Modelling of the Ocean (NEMO) with the 3DVar NEMOVAR system used for the assimilation of sea surface temperature data. This is coupled to the European Regional Seas Ecosystem Model (ERSEM), developed at Plymouth Marine Laboratory (PML). ERSEM based models have been used operationally to forecast biogeochemistry in the region for a number of years.

Spatial resolution 0.11 degree × 0.07 degree

Vertical coverage from -5500.0 m to 0.0 m

Temporal resolution Daily mean

Update frequency Daily

Production unit NWS-METOFFICE-EXETER-UK

DATASET #3

Product identifier **NORTHWESTSHELF_ REANALYSIS_ BIO_ 004_ 011**

Link `http://marine.copernicus.eu/services-portfolio/access-to-products/?option=com_csw&view=details&product_id=NORTHWESTSHELF_REANALYSIS_BIO_004_011` (last accessed on 10-06-2018)

Short description The reanalysis covers the period January 1985 until July 2014 and is based upon the Forecasting Ocean Assimilation Model 7km Atlantic Margin Model (FOAM AMM7).This is a hydrodynamic model of the North West European shelf forced at the surface by ERA-interim winds, atmospheric temperature, and precipitation fluxes. Horizontal boundary conditions were provided by the NOC global reanalysis prior to 1989 and by the GloSea reanalysis thereafter. Boundary conditions in the Baltic sea came from the IOM-GETM. E-Hype data were used for river inputs. Hydrodynamic calculations were performed by the Nucleus for European Modelling of the Ocean (NEMO) system, while the 3DVar NEMOVAR system was used for the assimilation of sea surface temperature data. Physical outputs are provided both as monthly means and as daily 25 hour, de-tided, averages.

Spatial resolution 0.11 degree × 0.07 degree

Vertical coverage from -5500.0 m to 0.0 m

Temporal resolution Daily mean

Update frequency Daily

Production unit NWS-METOFFICE-EXETER-UK

DATASET #4

Product identifier **NORTHWESTSHELF_ ANALYSIS_ FORECAST_ BIO_ 004_ 002_ b**

Link http://marine.copernicus.eu/services-portfolio/access-to-products/?option=com_csw&view=details&product_id=NORTHWESTSHELF_ANALYSIS_FORECAST_BIO_004_002_b (last accessed on 10-06-2018)

Short description The Forecasting Ocean Assimilation Model 7km Atlantic Margin model (FOAM AMM7) is a coupled hydrodynamic-ecosystem model, nested in a series of one-way nests to the Met Office global ocean model. The hydrodynamics are supplied by the Nucleus for European Modelling of the Ocean (NEMO) with the 3DVar NEMOVAR system used for the assimilation of sea surface temperature data. This is coupled to the European Regional Seas Ecosystem Model (ERSEM), developed at Plymouth Marine Laboratory (PML). ERSEM based models have been used operationally to forecast biogeochemistry in the region for a number of years.

Spatial resolution 0.11 degree $\times$ 0.07 degree

Vertical coverage from -5500.0 m to 0.0 m

Temporal resolution Daily mean

Update frequency Daily

Production unit NWS-METOFFICE-EXETER-UK

Predator abundance datasets

Decapods (macrobenthos) datasets

Information on benthic decapods abundance (macrobenthos, number of individuals per sample) for the period 1978 - 2017 in the Belgian coastal area (51°20'N - 51°05'N, 03°12'E - 02°32'E) were obtained from the ICES Data Centre (`http://www.ices.dk/marine-data/`). These include analyses based on fisheries data, coastal benthos survey projects, and literature:

- ICES North Sea Benthos Survey (1986 - 2000) (Kröncke et al., 2011)
- North Sea Benthos Project 2000 (1999 - 2001) (Reiss et al., 2006)
- other monitoring projects and literature (1978 - 2017): Basford & Eleftheriou (1988), Eleftheriou & Basford (1989), Basford et al. (1993), Craeymeersch et al. (1997), Ghertsos et al. (2000), Heip & Craeymeersch (1995), Kirby & Beaugrand (2009), Kirby et al. (2009), Kröncke & Bergfeld (2003), Kunitzer et al. (1992), Lindley et al. (2010), Luczak et al. (2012), Reiss et al. (2007).

Decapods (plankton) datasets

Decapod planktonic larvae datasets for the period 1958 - 2009 period were obtained from the ICES Data Centre (`http://www.ices.dk/marine-data/`), and the Management Unit of the Mathematical Model of the North Sea (MUMM, at the RBINS, `http://www.bmdc.be/`), and European Environment Agency (EEA), `https://www.eea.europa.eu/`). These include data from the Continuous Plankton Recorder (CPR) survey (Batten et al., 2003), operating in the North Sea on a monthly basis since 1946 and representing the largest marine biological time series available (monthly abundance data of holozooplankton and merozooplanktonic larvae of decapods, and other invertebrate taxa).

Gull datasets

The number and location of breeding pairs along the Belgian coastlines for the four dominant sea gull species, the black-headed gull (*Larus ridibundus*, 1969 - 2007), lesser black-headed gull (*Larus fuscus graellsii*, 1985 - 2007), herring gull (*Larus argentatus*, 1960 - 2007) and common gull (*Larus canus*, 1975 - 2007), were obtained from the following literature: De Groote (2003), Engledow et al. (2001), Luczak et al. (2012), Seys et al. (1998), Spanoghe (1999), Stienen et al. (2002), Stuer (2002), Vermeersch & Anselin (2009), Vermeersch et al. (2007).

Dog whelks

Information on presence of the dog whelk *Nucella lapillus* was obtained from published literature (De Blauwe & D'Udekem d'Acoz, 2012) and reports (OSPAR, 2009).

References

Adams, D. C., Rohlf, F. J., & Slice, D. E. (2004). Geometric morphometrics: ten years of progress following the "revolution". *Italian Journal of Zoology*, *71*(1), 5–16. doi: 10.1080/11250000409356545

Barton, K. (2017). *MuMIn: Multi-Model Inference.* Retrieved 2019-03-19, from `https://CRAN.R-project.org/package=MuMIn`

Basford, D. J., & Eleftheriou, A. (1988). The benthic environment of the North Sea (56°to 61°N). *Journal of the Marine Biological Association of the United Kingdom*, *68*(01), 125–141. doi: 10.1017/S0025315400050141

Basford, D. J., Eleftheriou, A., Davies, I. M., Irion, G., & Soltwedel, T. (1993). The ICES North Sea benthos survey: the sedimentary environment. *ICES Journal of Marine Science*, *50*(1), 71–80. doi: 10.1006/jmsc.1993.1008

Bates, D., Mächler, M., Bolker, B., & Walker, S. (2015). Fitting linear mixed-effects models using lme4. *Journal of Statistical Software*, *67*(1), 1–48. doi: 10.18637/jss.v067.i01

Batten, S., Clark, R., Flinkman, J., Hays, G., John, E., John, A., ... Walne, A. (2003). CPR sampling: the technical background, materials and methods, consistency and comparability. *Progress in Oceanography*, *58*(2-4), 193–215. doi: 10.1016/J.POCEAN.2003.08.004

Becuwe, M. (1971). Het voorkomen van de Steenloper, *Arenaria interpres*, en de Paarse Strandloper, *Calidris maritima*, in België en Zeeuws-Vlaanderen (Nederland). *Le Gerfaut*, *61*, 175–223.

Bonhomme, V., Picq, S., Gaucherel, C., & Claude, J. (2014). Momocs : outline analysis using R. *Journal of Statistical Software*, *56*(13), 1–24. doi: 10.18637/jss.v056.i13

Bookstein, F. L. (1991). *Morphometric tools for landmark data: geometry and biology.* Cambridge: Cambridge University Press.

Brooks, M. E., Kristensen, K., van Benthem, K. J., Magnusson, A., Berg, C. W., Nielsen, A., ... Bolker, B. M. (2017). Modeling Zero-Inflated Count Data With glmmTMB. *bioRxiv*, 132753. doi: 10.1101/132753

Claude, J. (2008). *Morphometrics with R.* Springer.

Claude, J. (2013). Log-shape ratios, Procrustes superimposition, elliptic Fourier analysis: three worked examples in R. *Hystrix*, *24*(1), 94–102. doi: 10.4404/hystrix-24.1-6316

Craeymeersch, J., Heip, C., & Buijs, J. (1997). *Atlas of the North Sea Benthic Infauna, ICES Cooperative Research Report 218* (Tech. Rep.). ICES.

Crampton, J. S. (1995). Elliptic Fourier shape analysis of fossil bivalves: some practical considerations. *Lethaia*, *28*(2), 179–186. doi: 10.1111/j.1502-3931.1995.tb01611.x

Cribari-Neto, F., & Zeileis, A. (2010). Beta regression in R. *Journal of Statistical Software*, *34*(2), 1–24. doi: 10.18637/jss.v034.i02

De Blauwe, H., & D'Udekem d'Acoz, C. (2012). Voortplantende populatie van de Purperslak (*Nucella lapillus*)in België na meer dan 30 jaar afwezigheid (Mollusca, Gastropoda, Muricidae). *De Strandvlo*, *32*(4), 127–131.

De Groote, D. (2003). *Aantalsverloop, Verspreiding en Gedrag Vanwatervogels in en Rond de Ijzermonding te Nieuwpoort Inrelatie tot het Getij. M.Sc. thesis.* (Unpublished doctoral dissertation). University of Gent.

Eleftheriou, A., & Basford, D. (1989). The macrobenthic infauna of the offshore northern North Sea. *Journal of the Marine Biological Association of the United Kingdom*, *69*(01), 123–143. doi: 10.1017/S0025315400049158

Engledow, H., Spanoghe, G., Volckaert, A., Coppejans, E., Degraer, S., Vincx, M., & Hoffmann, M. (2001). *Onderzoek naar de Fysischekarakterisatie en de Biodiversiteit van Strandhoofden en Andereharde Constructies langs de Belgische Kust.* (Tech. Rep.). Gent: . Instituut voorNatuurbehoud, Brussel, Universiteit Gent.

EU Copernicus Marine Service. (2018). *Copernicus marine environment monitoring service - CMEMS.* Retrieved 2019-03-19, from `http://marine.copernicus.eu/`

Fox, J., & Weisberg, S. (2011). *An R Companion to Applied Regression.* Thousand Oaks, CA, USA: SAGE Publications.

Gaisford, S., Kett, V., & Haines, P. J. (2016). *Principles of Thermal Analysis and Calorimetry: Edition 2.* Cambridge, UK: Royal Society of Chemistry. doi: 10.1039/9781847551764

Ghertsos, K., Luczak, C., Dewarumez, J.-M., & Dauvin, J.-C. (2000). Influence of spatial scales of observation on temporal change in diversity and trophic structure of fine-sand communities from the English Channel and the southern North Sea. *ICES Journal of Marine Science*, *57*(5), 1481–1487. doi: 10.1006/jmsc.2000.0916

Giardina, C. R., & Kuhl, F. P. (1977). Accuracy of curve approximation by harmonically related vectors with elliptical loci. *Computer Graphics and Image Processing*, *6*(3), 277–285. doi: 10.1016/S0146-664X(77)80029-4

Haines, A. J., & Crampton, J. S. (2000). Improvements to the method of Fourier shape analysis as applied in morphometric studies. *Palaeontology*, *43*(4), 765–783. doi: 10.1111/1475-4983.00148

Haines, P. J. (2002). *Principles of Thermal Analysis and Calorimetry.* Cambridge, UK: Royal Society of Chemistry. doi: 10.1039/9781847551764

Heip, C., & Craeymeersch, J. A. (1995). Benthic community structures in the North Sea. *Helgoländer Meeresuntersuchungen*, *49*(1-4), 313–328. doi: 10.1007/BF02368359

ICES. (2004). *Report of the ICES Advisory Committee on Fishery Management and Advisory Committee on Ecosystems, 2004. ICES Advice. Volume 1, Number 2.* (Tech. Rep.). Copenhagen: Author.

IMERS. (2018). *Integrated Marine Environmental Readings and Samples. Flanders Marine Institute.* Retrieved 2018-02-09, from `http://www.vliz.be/vmdcdata/imers/imers.php`

Kirby, R. R., & Beaugrand, G. (2009). Trophic amplification of climate warming. *Proceedings of the Royal Society B*, *276*(1676), 4095–4103. doi: 10.1098/rspb.2009.1320

Kirby, R. R., Beaugrand, G., & Lindley, J. A. (2009). Synergistic effects of climate and fishing in a marine ecosystem. *Ecosystems*, *12*(4), 548–561. doi: 10.1007/s10021-009-9241-9

Kröncke, I., & Bergfeld, C. (2003). North sea benthos: a review. *Senckenbergiana maritima*, *33*(1-2), 205–268. doi: 10.1007/BF03043049

Kröncke, I., Reiss, H., Eggleton, J. D., Aldridge, J., Bergman, M. J., Cochrane, S., ... Rees, H. L. (2011). Changes in North Sea macrofauna communities and species distribution between 1986 and 2000. *Estuarine, Coastal and Shelf Science*, *94*(1), 1–15. doi: 10.1016/j.ecss.2011.04.008

Kuhl, F. P., & Giardina, C. R. (1982). Elliptic Fourier features of a closed contour. *Computer Graphics and Image Processing*, *18*(3), 236–258. doi: 10.1016/0146-664X(82)90034-X

Kunitzer, A., Basford, D., Craeymeersch, J. A., Dewarumez, J. M., Dorjes, J., Duineveld, G. C. A., ... de Wilde, P. A. J. (1992). The benthic infauna of the North Sea: species distribution and assemblages. *ICES Journal of Marine Science*, *49*(2), 127–143. doi: 10.1093/icesjms/49.2.127

Kuznetsova, A., Brockhoff, P. B., & Christensen, R. H. B. (2017). lmerTest package: tests in linear mixed effects models. *Journal of Statistical Software*, *82*(13), 1–26. doi: 10.18637/jss.v082.i13

Lindley, J. A., Beaugrand, G., Luczak, C., Dewarumez, J.-M., & Kirby, R. R. (2010). Warm-water decapods and the trophic amplification of climate in the North Sea. *Biology letters*, *6*(6), 773–776. doi: 10.1098/rsbl.2010.0394

Luczak, C., Beaugrand, G., Lindley, J. A., Dewarumez, J.-M., Dubois, P. J., & Kirby, R. R. (2012). North Sea ecosystem change from swimming crabs to seagulls. *Biology Letters*, *8*(5), 821–824. doi: 10.1098/rsbl.2012.0474

OSPAR. (2009). *Background Document for Nucella lapillus (Dog whelk) Biodiversity Series* (Tech. Rep.). Author.

Pinheiro, J., Bates, D., DebRoy, S., Sarkar, D., & R Core Team. (2017). *nlme: linear and nonlinear mixed effects models.*

R Core Team. (2016). *R: A language and environment for statistical computing.* Retrieved from `https://www.r-project.org/`

Reiss, H., Meybohm, K., & Kröncke, I. (2006). Cold winter effects on benthic macrofauna communities in near- and offshore regions of the North Sea. *Helgoland Marine Research*, *60*(3), 224–238. doi: 10.1007/s10152-006-0038-3

Reiss, H., Wieking, G., & Kroencke, I. (2007). Microphytobenthos of the Dogger Bank: a comparison between shallow and deep areas using phytopigment composition of the sediment. *Marine Biology*, *150*, 1061 – 1071.

Rohlf, F. J., & Archie, J. W. (1984). A comparison of Fourier methods for the description of wing shape in mosquitoes (Diptera: Culicidae). *Systematic Zoology*, *33*(3), 302. doi: 10.2307/2413076

Rohlf, F. J., & Marcus, L. F. (1993). A revolution in morphometrics. *Trends in Ecology & Evolution*, *8*(4), 129–132. doi: 10.1016/0169-5347(93)90024-J

Ruppert, D., Wand, M. P., & Carroll, R. J. (2003). *Semiparametric Regression.* Cambridge: Cambridge University Press.

Seys, J., VanWaeyenberge, J., Devos, K., Meire, P., & Kuijken, E. (1998). The recent expansion of breeding gulls along the Belgian North Sea coast. *Sula*, *12*(4), 209–216.

Simpson, G. (2018). *tsgam: Utilities for Working with GAMs Fitted to Time Series.* Retrieved 2019-03-19, from `https://github.com/gavinsimpson/tsgam`

Spanoghe, G. (1999). *Aantallen en Verspreiding, gedrag en Habi-Tatkeuze van Meeuwen (Laridae) aan de Vlaamse Kust in Hetwinterhalfjaar. M.Sc. thesis* (Unpublished doctoral dissertation). University of Ghent.

Stienen, E., Van Waeyenberge, J., & Vercruijsse, J. (2002). Zilvermeeuw *Larus argentatus* en Kleine Mantelmeeuw *Larus fuscus* als broedvogels in Vlaanderen. *Nattur Oriolus*, *68*(3), 104–110.

Stuer, V. (2002). *Trofische Interacties in Relatie tot Zwinnen Vande Belgische kust (Schipgatduinen Koksijde): Epibenthos Enavifauna. M.Sc. thesis* (Unpublished doctoral dissertation). University of Gent.

Telesca, L., Michalek, K., Sanders, T., Peck, L. S., Thyrring, J., & Harper, E. M. (2018). Blue mussel shell shape plasticity and natural environments: a quantitative approach. *Scientific Reports*, *8*(1), 2865.

Thompson, D. W. (1917). *On Growth and Form.* Cambridge: Cambridge University Press.

Vermeersch, G., & Anselin, A. (2009). *Broedvogels in Vlaanderen in 2006-2007* (Tech. Rep.). Instituut voor Natuur.

Vermeersch, G., Anselin, A., & Devos, K. (2007). *Bijzondere Broedvogels in Vlaanderen in de periode 1994-2005* (Tech. Rep.). Brussels: Instituut voor Natuur.

Warmose, T., Backeljau, T., & De Bruyn, L. (1988). The littorinid fauna of the Belgian coast (Mollusca, Gastropoda). *Bulletin De L'institut Royal Des Sciences Naturelles De Belgique*, *58*, 51–70.

Wickham, H. (2016). *Ggplot2 : Elegrant Graphics for Data Analysis.* New York, NY, USA: Springer.

Wood, S. (2017). *Generalized Additive Models : an Introduction with R, Second Edition.* London: Chapman & Hall/CRC.

Wood, S., & Scheipl, F. (2017). *gamm4: Generalized Additive Mixed Models using 'mgcv' and 'lme4'.*

Zaremba, C. M., Morse, D. E., Mann, S., Hansma, P. K., & Stucky, G. D. (1998). Aragonite-hydroxyapatite conversion in gastropod (Abalone) nacre. *Chemistry of Materials*, *10*(12), 3813–3824. doi: 10.1021/cm970785g
